# Small extracellular vesicles convey the stress-induced adaptive responses of melanoma cells

**DOI:** 10.1038/s41598-019-51778-6

**Published:** 2019-10-25

**Authors:** Maria Harmati, Edina Gyukity-Sebestyen, Gabriella Dobra, Laszlo Janovak, Imre Dekany, Okay Saydam, Eva Hunyadi-Gulyas, Istvan Nagy, Attila Farkas, Tibor Pankotai, Zsuzsanna Ujfaludi, Peter Horvath, Filippo Piccinini, Maria Kovacs, Tamas Biro, Krisztina Buzas

**Affiliations:** 1 Laboratory of Microscopic Image Analysis and Machine Learning, Institute of Biochemistry, Biological Research Centre of the Hungarian Academy of Sciences, Szeged, Hungary; 2 Doctoral School of Interdisciplinary Medicine, Faculty of Medicine, University of Szeged, Szeged, Hungary; 3 Interdisciplinary Excellence Centre, Department of Physical Chemistry and Materials Science, University of Szeged, Szeged, Hungary; 4 Department of Pediatrics, University of Minnesota, Minneapolis, USA; 5 Laboratory of Proteomics Research, Institute of Biochemistry, Biological Research Centre of the Hungarian Academy of Sciences, Szeged, Hungary; 6 Sequencing Platform, Institute of Biochemistry, Biological Research Centre of the Hungarian Academy of Sciences, Szeged, Hungary; 7 Laboratory of Microbial Genomics, Institute of Plant Biology, Biological Research Centre of the Hungarian Academy of Sciences, Szeged, Hungary; 8 Department of Biochemistry and Molecular Biology, Faculty of Science and Informatics, University of Szeged, Szeged, Hungary; 9 Department of Oral Biology and Experimental Dental Research, Faculty of Dentistry, University of Szeged, Szeged, Hungary; 10 Institute for Molecular Medicine Finland, University of Helsinki, Helsinki, Finland; 11 Laboratorio di Bioscienze, Istituto Scientifico Romagnolo per lo Studio e la Cura dei Tumori (IRST) IRCCS, Meldola, Italy; 12 Department of Immunology, Faculty of Medicine, University of Debrecen, Debrecen, Hungary; 13 Hungarian Centre of Excellence for Molecular Medicine, Szeged, Hungary

**Keywords:** Cancer, Cell biology

## Abstract

Exosomes are small extracellular vesicles (sEVs), playing a crucial role in the intercellular communication in physiological as well as pathological processes. Here, we aimed to study whether the melanoma-derived sEV-mediated communication could adapt to microenvironmental stresses. We compared B16F1 cell-derived sEVs released under normal and stress conditions, including cytostatic, heat and oxidative stress. The miRNome and proteome showed substantial differences across the sEV groups and bioinformatics analysis of the obtained data by the Ingenuity Pathway Analysis also revealed significant functional differences. The *in silico* predicted functional alterations of sEVs were validated by *in vitro* assays. For instance, melanoma-derived sEVs elicited by oxidative stress increased Ki-67 expression of mesenchymal stem cells (MSCs); cytostatic stress-resulted sEVs facilitated melanoma cell migration; all sEV groups supported microtissue generation of MSC-B16F1 co-cultures in a 3D tumour matrix model. Based on this study, we concluded that (i) molecular patterns of tumour-derived sEVs, dictated by the microenvironmental conditions, resulted in specific response patterns in the recipient cells; (ii) *in silico* analyses could be useful tools to predict different stress responses; (iii) alteration of the sEV-mediated communication of tumour cells might be a therapy-induced host response, with a potential influence on treatment efficacy.

## Introduction

Exosomes are small (30–200 nm)^1^ endosome-derived vesicles that are actively secreted into the extracellular environment from most cell types. Initially, exosomes were proposed to eliminate cellular waste, but it has been proven that they also play a key role in the intercellular communication between adjacent as well as distal cells through the horizontal transfer of lipids, proteins and nucleic acids. Over the past three decades, exosomes have surged to the forefront of cell biology research, and recently an increasing body of evidence indicates that this exosomal communication is a deliberate and highly orchestrated process. Clinical relevance of exosomes is also considerable, since they are associated with numerous physiological and pathological conditions, including cancer diseases^2^.

Tumours are not just insular masses of proliferating cancer cells; they are also complex tissues composed of cellular components, such as mesenchymal stem cells (MSCs), cancer-associated fibroblasts (CAFs), endothelial cells, immune cells as well as extracellular matrix (ECM) components, which establish the so-called tumour microenvironment (TME) surrounding the tumour cells. The TME does not only surround the tumour cells, it also actively contributes to tumour progression, which requires a continuous paracrine communication^3,4^. One of the possible candidates for intercellular communication might be exosomes, since several recent papers have emphasized the mediating role of exosomes in the tumour macro- and microenvironment^4–6^. Upon contact with recipient cells, tumour-derived exosomes alter their phenotypic and functional properties conveying molecular and genetic messages^7^.

Since malignant melanoma is one of the most aggressive cancers, the B16F1 mouse melanoma cell line was chosen as a tumour cell model for this study. Melanoma cells potentially disseminate from a relatively small primary tumour and form metastases in multiple sites, including the lung, liver, brain, bone and lymph nodes^8^. This high metastatic potential can be explained by the unique features of melanoma. For instance, melanoma cells are mesenchymal in nature ensuring that a larger percentage of cells can act as stem cells with self-renewal capacity. They share many antigens with vascular endothelial cells (vasculogenic mimicry) which enables them to survive in the circulation, and increases their migration and invasion capacity as well. Furthermore, melanoma-derived extracellular vesicles also have a crucial role in the rapid tumour progression^8–12^. They are capable to induce a tumour-favourable phenotype in the EV-recipient cells in the TME^13^ and the metastatic sites^14,15^. For instance, after re-education by exosomes, MSCs may promote tumour growth and metastasis^16,17^; fibroblast^18^ and endothelial cells^19^ can promote angiogenesis. There are also a few papers about intrinsic stresses, such as low pH^20^- or hypoxia^21^-induced alterations of melanoma exosomes, and their ability to transfer drug resistance^22^. However, different extrinsic stress-elicited changes have not been elucidated yet.

Using a unique approach, this study compared changes in the vesicular information transfer of melanoma cells under different inducible stress conditions. Exosomes are complex information packages, and we consider them as message delivering units with specific molecular patterns, rather than putting emphasis on a few exosomal signal molecules. Following the guideline recommended by ISEV (International Society for Extracellular Vesicles), called MISEV2018 (Minimal information for studies of extracellular vesicles 2018)^23^, we refer to the isolated vesicles based on their size by using the term ‘small extracellular vesicles’ (sEVs), even though their exosomal characteristics are demonstrated.

To gain insights into the plasticity and role of sEVs under suboptimal conditions, we investigated cytostatic, heat and oxidative stress-induced alterations of the B16F1 mouse melanoma cell-derived sEVs. Clinical relevance of this study is highlighted by previous papers, since chemo- or radiotherapy were shown to increase the amount of circulating tumour-derived EVs^24,25^. Although chemotherapy provides long-term clinical benefits to patients, it may induce tumour-promoting host responses as well^26^. Furthermore, Keklikoglou *et al*. have shown that paclitaxel and doxorubicin elicit the production of pro-metastatic breast cancer-derived EVs^27^. Hyperthermia treatment involves increasing the target site temperature to induce thermic stress, which results in cancer cell cytotoxicity and immune response stimulation via immune cell activation. Therefore, hyperthermia may enhance the therapeutic efficacy in combined therapies^28,29^, but its impact on tumour exosome-mediated intercellular communication has not been described yet. To induce oxidative stress, we used Ag-TiO_2_ photocatalyst particles, which represent a high potential for therapeutic applications through antibacterial^30,31^, antifungal^32^ and anticancer^33^ activities. However, the effects of Ag-TiO_2_-based therapies on the vesicular communication have not been investigated yet.

## Results and Discussion

**Figure 1:**
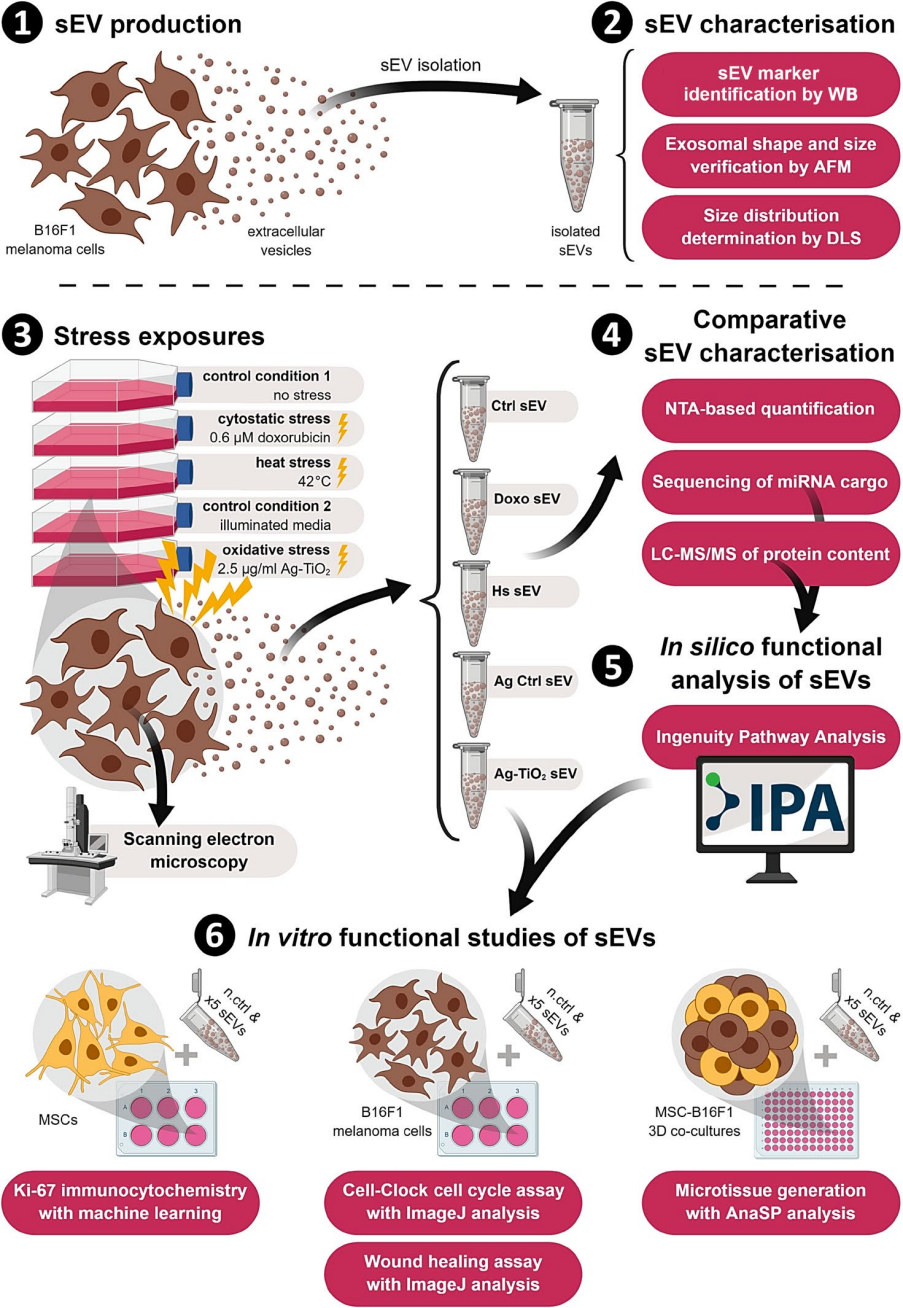
Schematic illustration of the experimental workflow in six steps. B16F1 mouse melanoma cell-derived sEVs were isolated and characterised by Western Blot (WB), atomic force microscopy (AFM) and dynamic light scattering (DLS). Then B16F1 cultures were treated in five different ways, and 72 h supernatants were harvested for sEV isolation. Vesicle samples were then analysed by nanoparticle tracking analysis (NTA) to determine the number of released sEVs, sequencing and LC-MS/MS to describe their miRNome and proteome. Ingenuity Pathway Analysis (IPA) was used to analyse data and predict the functional differences between sEV groups. This *in silico* predictions were tested *in vitro* on mesenchymal stem cell (MSC) and melanoma cell cultures and MSC-B16F1 3D co-cultures as well using Ki-67-specific immunocytochemistry, Cell-Clock cell cycle assay, wound healing assay, and 3D hanging drop technology. Abbreviation: n.ctrl-negative control. Figure was created with BioRender.com.

In order to study the adaptive sEV-mediated communication under microenvironmental stress, we investigated cytostatic, heat and oxidative stress-induced alterations of the B16F1 mouse melanoma cell-derived sEVs. After verification the exosomal characteristics of our sEV isolates, we optimized the cytostatic and oxidative stress treatment conditions by proliferation assay and established the protocol for heat stress based on literature data^34^ to expose the melanoma cells to sublethal stress conditions. Then, for the sEV production, we cultured the B16F1 cells under five different conditions in EV-depleted FBS-containing media; control cultures (Ctrl) received culture medium, cytostatic stressed cultures (Doxo) were treated with 0.6 µM doxorubicin, heat stressed cultures (Hs) were incubated at 42°C for 3 × 2 h, oxidative stressed cultures (Ag-TiO_2_) were treated with 2.5 µg/ml light-induced Ag-TiO_2_, and as a control of the oxidative stress (Ag Ctrl), additional cultures were treated with illuminated media (Table 1). After a 72 h stress exposure period of B16F1 cultures, sEVs were isolated from their supernatants, quantified by nanoparticle tracking analysis (NTA) and analysed by SOLiD sequencing and LC-MS/MS to determine the miRNome and proteome of sEVs. Functional differences between sEV groups were predicted first *in silico* using the Ingenuity Pathway Analysis (IPA) based on the protein and miRNA data, and then verified by *in vitro* experiments targeting tumour-related cellular functions, such as Ki-67 expression, cell cycle dynamics, migration capacity and microtissue generation of the recipient cells (Fig. 1).

**Table 1:** Treatment schedule of tumour cell cultures and the isolated sEV groups.

Conditions	Control 1	Cytostatic stress	Heat stress	Control 2	Oxidative stress
sEV donor cell cultures	Ctrl	Doxo	Hs	Ag Ctrl	Ag-TiO_2_
Treatment	—	0.6 μM doxorubicin	3 × 2 h at 42°C	illuminated medium	light-induced 2.5 μg/ml Ag-TiO_2_
Released sEV groups	Ctrl sEV	Doxo sEV	Hs sEV	Ag Ctrl sEV	Ag-TiO_2_ sEV

Our oxidative stress model is based on the photocatalytic activity of the Ag-TiO_2_ particles^31,35^. During the process of photocatalysis under appropriate (exciting) wavelength, reactive hydroxyl radicals (OH·) are produced, which are primarily responsible for photooxidation of organic materials or inactivating bacteria^36^. Hydroxyl radicals are the most reactive oxygen species and cause irreversible DNA damages which could lead to DNA degradation in bacteria^36^. In our previous work, the amount of reactive hydroxyl radicals formed on Ag-TiO_2_ particles was determined by the hydrogen peroxide-induced luminol-dependent chemiluminescence reaction^30^. It was presented that concentration of the Ag-TiO_2_-produced OH· radicals was equivalent to 0.33 mM H_2_O_2_ after 20 min visible light illumination.

### Descriptive statistics of sEVs released under different microenvironmental conditions

#### Isolated EVs fulfil the minimal experimental requirements for small extracellular vesicles (sEVs**)**

First, to fulfil the minimal experimental requirements for extracellular vesicles, suggested in the MISEV2018^23^, we characterised the B16F1 cell-derived extracellular vesicles isolated from conditioned media by differential filtration and ultracentrifugation. Presence of the vesicles in the sEV isolates was verified by atomic force microscopy (AFM), and size distribution of the isolated vesicle population was described by dynamic light scattering (DLS) with a Z-average of 78 nm. EV markers, such as CD63 and CD9 (transmembrane proteins), HSP70, Alix and TSG101 (cytosolic proteins), Calnexin (negative sEV marker) were investigated in the vesicle isolates and the donor cell lysates by Western blot (Supplementary Fig. S1).

#### Vesicle production of melanoma cells is elevated under stress conditions

**Figure 2:**
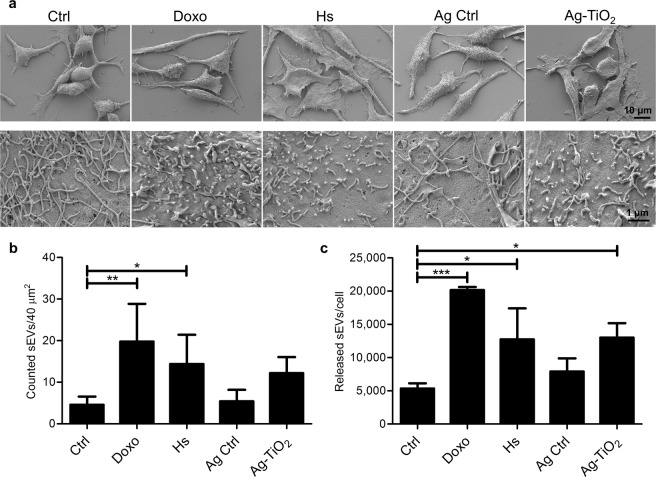
Microenvironmental stress factors resulted in morphological changes and elevated vesicle production of melanoma cells. (**a**) Scanning electron micrograph of the differently treated melanoma cells. The top row of pictures was taken in 1,500 × magnification showing the different cell morphology after 24 h treatments. The bottom row of pictures was taken in 20,000 × magnification showing the distinct cell surface structures. (**b**) The number of counted exosome-sized vesicles on the surface of cells using ImageJ (n = 5). (**c**) Number of released vesicles/cell based on NanoSight measurements (n = 3). Each bar represents mean + SD; *p < 0.05, **p < 0.01 and ***p < 0.001 indicate statistical significance.

Scanning electron microscopy (SEM) revealed spectacular morphological changes of the B16F1 cells in each stressed group (Doxo, Hs and Ag-TiO_2_) 24 h after treatments (Fig. 2a, top panels). Taking advantage of the high magnification capacity of SEM, we were able to observe the surface structures of the cells as well (Fig. 2a, bottom panels). At a 20,000 × magnification, we discovered spherical, exosome-sized vesicles, which were present in higher numbers on the stressed cells compared to the untreated Ctrl cells (p_Doxo_ = 0.00297, p_Hs_ = 0.03928, n = 5; Fig. 2b).

Then, we isolated sEVs from conditioned media of the five groups of cell cultures and quantified by the NTA-based NanoSight Analysis. There was a significant increase in vesicle number per cell in the Doxo (20.2 ± 0.4 × 10^3^; p = 0.00021) and the Hs (12.7 ± 3.8 × 10^3^; p = 0.03006) groups compared to the Ctrl one (5.35 ± 0.7 × 10^3^). The Ag-TiO_2_-treated cultures produced 13 ± 1.8 × 10^3^ sEVs per cell, while production of the Ag Ctrl group was only 7.9 ± 1.6 × 10^3^ sEVs per cell (n = 3, Fig. 2c). At the same time, stress conditions did not affect the size distribution of sEVs.

Other reports also demonstrated that cells, including tumour cells, release a higher amount of exosomes in response to different types of stresses^37^, such as hypoxia^38^, acidosis^39^, oxidative stress^40^, thermal stress^40^, radiation^41^ and cytotoxic drugs^42–44^.

#### Concentration of the encapsulated doxorubicin under cytostatic stress is less than 10% of median lethal dose

To reveal if doxorubicin, used for cytostatic stress in 0.6 µM concentration, could be encapsulated into the vesicles, Doxo sEV isolates were analysed by fluorescence spectroscopy. Using the Ctrl sEVs as a background, the calculated doxorubicin concentration of the Doxo sEVs was 14.735 nM.

Based on the measurements, the Doxo sEV suspensions – used for treatment of recipient cells – contained only 8 ng/ml doxorubicin. Therefore, it cannot be excluded that sEVs may transfer doxorubicin to the recipient cells, but the doxorubicin content of Doxo sEVs is less than 10% of the median lethal dose (LD50 = 100 ng/ml) for mouse melanoma cells^45^.

#### Encapsulation of Ag-TiO_2_ nanoparticles into sEVs cannot be proven

To investigate the possibility of Ag-TiO_2_ encapsulation into sEVs, we measured the size distribution of the nanoparticles by DLS. The mean particle size was around 255 nm during the whole studied time interval (Supplementary Fig. S2). This means that the initial Ag-TiO_2_ photocatalyst particles form aggregates because the primer size of the Ag-TiO_2_ particles is around 25 nm as seen in the TEM image in our previous paper^46^. Thus, DLS measurements did indicate particle aggregation in the used medium; however, it also can be seen that the size of these particle aggregates did not change during the experiments. These results suggest that the Ag-TiO_2_ particles cannot be transferred into the recipient cells in this aggregated form and they cannot contaminate the sEV isolates, since particles over 220 nm were eliminated during the isolation process.

We also tried to detect disaggregated Ag-TiO_2_ nanoparticles in the sEV isolates using chemiluminescence (CL) method and transmission electron microscopy (TEM). After photoirradiation of the Ag-TiO_2_, generated ROS can be detected by adding luminol, which emit light upon oxidation^30^. We compared the CL intensity of Ag-TiO_2_ sEV lysates to an Ag-TiO_2_ calibration curve prepared in Ag Ctrl sEV lysate, but it was under the detection limit (0.25 µg/ml; Supplementary Fig. S2). Moreover, on TEM images we could not observe any internal structures of the sEVs or contaminating electron-dense nanoparticles in the Ag-TiO_2_ sEV isolate (Supplementary Fig. S2).

#### miRNome and proteome of the melanoma sEVs strongly depend on the microenvironmental conditions of the donor cells

Exosomes deliver a wide range of RNAs and proteins to convey messages to the recipient cells. Their molecular content correlates with the type and state of the donor cell^2^. In this study, we compared the miRNA and protein patterns of the sEV groups to show the influence of the microenvironment on these information packages.

**Figure 3:**
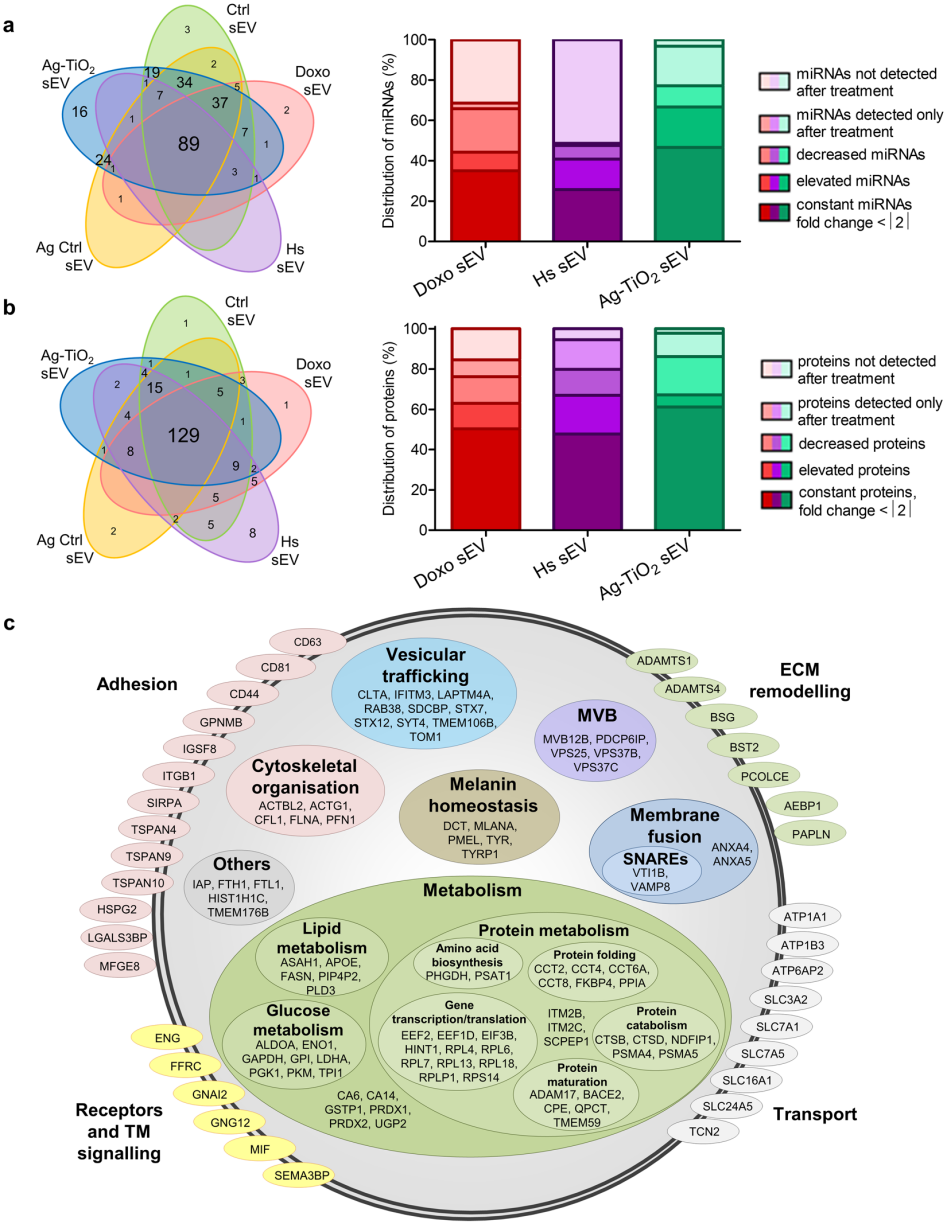
Stress factors caused unique molecular patterns of the melanoma-derived sEVs. (**a**,**b**) Results of the miRNA sequencing and whole proteome analysis by LC-MS/MS. Venn diagrams show the number of common and unique molecules of the different sEVs. Stacked bar graphs show the distribution of sEV molecules based on their changes compared to the appropriate control. (**c**) Classification of the common proteins for each sEV group based on their function and localisation in vesicles.

A total of 254 miRNAs were identified by SOLiD sequencing with more than ten read counts; 35.04% of these miRNAs were detected in each sEV group, while 6.30%, 1.18% and 0.79% were exclusively detected in the Ag-TiO_2_, Ctrl and Doxo sEVs, respectively. Hs and Ag Ctrl sEV-specific miRNAs were not found (Fig. 3a, left panel). The proportion of the over- and underrepresented miRNAs under stress conditions was the highest in the Ag-TiO_2_ sEVs, where 53.41% of the detected miRNAs showed more than twofold changes compared to the corresponding control (Fig. 3a, right panel). Results of the sequencing data were validated by qPCR on three major miRNAs of this study, *i.e*. mmu-miR-16-5p, mmu-miR-125b-5p and mmu-miR-29a-3p (Supplementary Fig. S3).

Using LC-MS/MS, a total of 216 proteins were detected with three or more peptides; 59.72% of these proteins were common to all sEV groups, and only a few unique proteins were found (Fig. 3b, left panel). Eight Hs sEV-specific proteins were identified; half of these had a chaperone function (HSBP1, SERPINH1, CCT5, CLU). The major impact of heat stress on the protein composition of sEVs was also evidenced by the high proportion of the over- and underrepresented proteins (52.21%) compared to the Ctrl sEV group. Cytostatic stress also resulted in dramatic and distinct changes in the protein content of vesicles. For instance, the proportion of proteins, which decreased below the detectable level was the highest (15.34%) in Doxo sEVs (Fig. 3b, right panel). Many of the commonly found proteins have a function in vesicular trafficking, membrane fusion or MVB biology which suggests they have a role in exosome biogenesis. There are also melanocyte-specific molecules, such as some melanin biosynthesis elements (DCT, MLANA, PMEL, TYR or TYRP1), and we found a several metabolism-, cytoskeletal organisation-, extracellular matrix remodelling-related proteins, adhesion molecules, receptors and transporters, which may have a crucial role in tumour progression (Fig. 3c). Proteomics data were validated by Western blot, where a vesicular marker, the HSP70 and the MLANA showed similar signal intensity patterns to the LC-MS/MS data (Supplementary Fig. S4).

Our findings about the molecular cargo of sEVs are in accordance with previous studies, which also showed changes in the exosomal content upon exposure of the donor cells to external stimuli and stress conditions^41,44,47,48^. Furthermore, Peinado *et al*.^17^ and Lazar *et al*.^49^, analysing protein composition of different melanoma cell line-derived exosomes, also found melanocyte-specific proteins, transmembrane proteins, such as tetraspanins, transporters and receptors as well as MVB and endosomal pathway-related proteins, for instance ESCRT-associated proteins, annexins, cytoskeletal and small GTP-binding proteins^17,49^.

### Comprehensive *in silico* analysis of functional differences between sEV groups

Since the exosomal cargo is a complex information package containing a large number and wide variety of molecules, it may act on several biological processes in the recipient cells. In this study, we aimed to identify these biological processes even for normal (Ctrl) B16F1 sEVs and also for the stress-exposed cell-derived ones. We performed bioinformatics analyses to interpret the biological context of the obtained miRNA and protein data applying the IPA. This software is based on computer algorithms that analyse the functional connectivity of the molecules using the Ingenuity Knowledge Base. For these *in silico* analyses, we set the confidence level to ‘Experimentally observed’ that enables literature data-based analysis, but not unproven predictions. Phrases between apostrophes are ‘IPA-specific terms’ in this paper.

**Figure 4:**
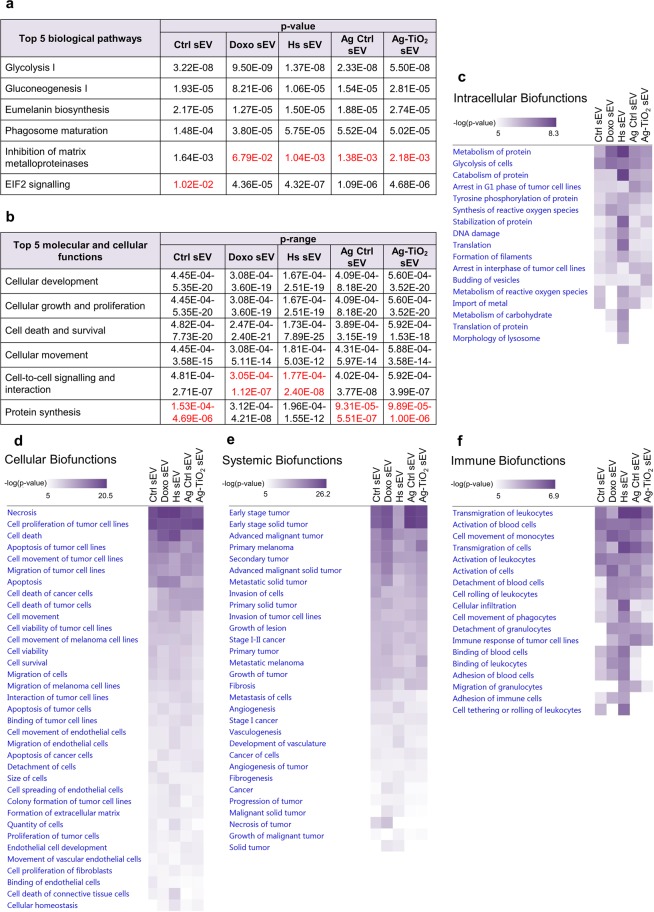
IPA showed that sEVs may influence many biological pathways and functions with different significance via their miRNA and protein content. (**a**) ‘Top 5 canonical pathways’ for each sEV group. Red values label the significance of pathways, which were not included in the Top 5. (**b**) ‘Top 5 molecular and cellular functions’ for each sEV group. Red values label the significance ranges of functions, which were not included in the Top 5. (**c**–**f**) Heatmaps from the ‘Comparison analysis’ of the molecular content of vesicles. Relevant ‘Biofunctions’ with -log(p-value) > 5 were organised into four groups, namely intracellular, cellular, systemic and immune processes.

Using the ‘Core analysis’ feature, we found a huge overlap in the ‘Top 5 canonical pathways’ between sEV groups. Namely, ‘Gycolysis I’, ‘Gluconeogenesis I’, ‘Eumelanin Biosynthesis’ and ‘Phagosome maturation’ was listed in all five cases, while ‘Inhibition of matrix metalloproteinases’ (MMPs) was only found in Ctrl sEVs, and ‘EIF2 signalling’ was listed in the four other sEV groups (Fig. 4a). As it was shown in the previous section, beside the remarkable differences, sEVs contain many donor cell-specific molecules, which helps to interpret these results. For instance, the Warburg effect, which means, that cancer cells may prefer metabolism via aerobic glycolysis rather than oxidative phosphorylation^50,51^ gives a possible explanation for the strong presence of glycolysis and glyconeogenesis-related molecules in sEVs. Since the investigated B16F1 is a melanin-producing cell line, the reason for the presence of the eumelanin biosynthesis-related molecules is obvious. Vesicular processes of exosome biogenesis^52^ account for the high number of phagosome maturation-related molecules in the isolated vesicles. Presence of the MMP- and EIF2 signalling-related molecules in the sEVs can also be explained by the tumour cell origin^53–55^.

‘Top 5 molecular and cellular functions’ also showed similarities across the sEV groups. ‘Cellular development’, ‘Cellular growth and proliferation’, ‘Cell death and survival’ and ‘Cellular movement’ were shared between each sEV group; ‘Protein synthesis’ was listed in Doxo and Hs sEVs and ‘Cell-to-cell signalling and interaction’ in Ctrl, Ag Ctrl and Ag-TiO_2_ ones (Fig. 4b). All of these cellular functions are involved in tumour progression, which highlights the role and diverse effects of vesicles in recipient cell reprogramming in the tumour matrix and the metastatic sites^4–7,56–58^.

Performing ‘Comparison analysis’ in the IPA, we built a heatmap of the melanoma-related ‘Diseases and Biofunctions’, significantly influenced by any sEV group (-log(p-value) > 5). This *in silico* analysis revealed that the sEVs may play a role not only in intracellular and cellular, but in systemic and immunological processes as well (Fig. 4c–f). Focusing on the activation and inhibitory effects, we identified many ‘Biofunctions’, which may be regulated differently by the sEVs, highlighting the role of the releasing conditions in the vesicular communication of melanoma cells. In order to prove the tumour-associated functional differences between sEV groups, some ‘Biofunctions’ related to stem cell proliferation, cell cycle, migration of tumour cells and aggregation of cells were selected for *in vitro* investigations.

### *In silico* predictions-based *in vitro* analyses of sEV-induced cellular responses in tumour matrix cells

In regulation-focused examinations using the LC-MS/MS and SOLiD sequencing data, the ‘Grow tool’ of IPA enabled to identify the interacting vesicular molecules for the investigated ‘Biofunctions’. Then, the ‘Molecule Activity Predictor’ (MAP) feature of IPA predicted their overall regulatory effects for each sEV group. Following these *in silico* studies, predicted alterations of the sEV-induced cell responses were analysed experimentally by *in vitro* methods. Proliferation, cell cycle dynamics, migration capacity and microtissue generation of the sEV recipient cells were investigated by Ki-67 immunocytochemistry, Cell-Clock cell cycle assay, wound healing assay and hanging drop technology, respectively.

#### Ag-TiO_2_ sEVs facilitate proliferation of mesenchymal stem cells

**Figure 5:**
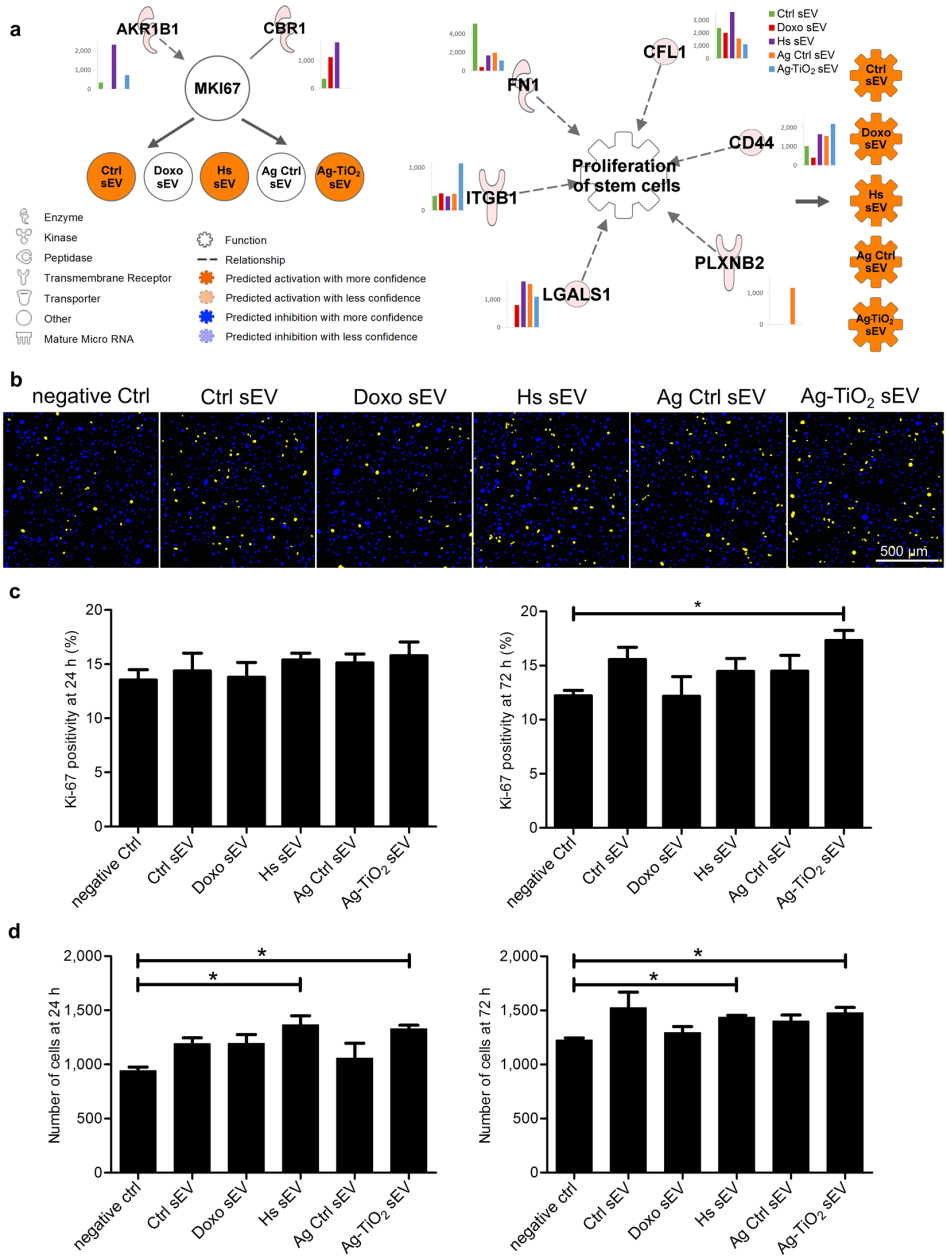
Stress-exposed melanoma cell-derived vesicles affected the proliferation of MSCs. (**a**) IPA predictions for the regulatory effects of sEV molecules on Ki-67 expression and ‘Proliferation of stem cells’. Networks show every upstream regulator proteins accompanied by a bar graph, which represents the normalised expression values of the molecule for each sEV group. Coloured symbols, named as the sEV groups, display the expected regulation changes of the analysed ‘Molecule’ and ‘Biofunction’ upon exposure to the vesicles. (**b**,**c**) Evaluation of the Ki-67-specific immunocytochemistry using an image analysis and machine learning software. (**b**) Images are representatives of the classified ones. Yellow and blue dots show the Ki-67 positive and negative nuclei, respectively. (**c**) Bar graphs show percentages of the Ki-67 positive cells 24 h (left panel) and 72 h (right panel) after sEV exposures. Each bar represents mean + SD (n = 4). (**d**) Cell numbers of the sEV-exposed cells after 24 h and 72 h incubation time. Bar graphs represent mean + SD values (n = 3), *p < 0.05 indicates statistical significance.

As an important element of the TME, the MSCs can be targeted by the tumour-derived extracellular vesicles. Therefore, we investigated the effects of the different sEVs on stem cells. First, the *in silico* analyses predicted activation of Ki-67 expression for the Ctrl, Hs and Ag-TiO_2_ sEVs, and activation of ‘Proliferation of stem cells’ for each sEV group. These predictions suggest that after internalisation by stem cells, each of the investigated sEV groups may induce cell divisions and three of them could result in Ki-67 upregulation, if the delivered vesicular cargo is active in the recipient cells (Fig. 5a). According to the IPA analyses, the key B16F1 vesicular regulator of the Ki-67 expression may be the aldo-keto reductase family 1 member B1 (AKR1B1), which is known to be involved in glucose metabolism, osmoregulation, detoxication of lipid aldehydes^59^, oxidative stress signalling, activation of NF-κB and expression of adhesion molecules, such as ICAM or VCAM. It has recently been shown that inhibition of AKR1B1 prevented proliferation and expression of Ki-67 in the human umbilical vein endothelial cells (HUVEC)^60^. The IPA-predicted activation of ‘Proliferation of stem cells’ might be enhanced for instance by fibronectin (FN1)^61^, which was previously described by Sharma *et al*. to increase the growth of embryonic stem cells^61^ (Fig. 5a).

To test the differences in the Ki-67 regulation across sEV groups, we treated MSC cultures with 200 µg/ml sEV suspensions or PBS buffer as a negative control. After 24 h or 72 h of vesicle exposures, the Ki-67 expression was investigated by immunocytochemistry. For the quantitative evaluation of the experiment, the Operetta high-content imaging system and an image analysis and machine learning software (SCT Analyzer 1.0)^62^ was applied, which enabled to analyse almost 1.6 × 10^5^ cells within a few hours. Our computer-assisted image analysis pipeline was comprised of cell segmentation, feature extraction and machine learning modules, where we had a training set with two classes for the Ki-67 positive and the negative cells. Compared to the negative control group, Ag-TiO_2_ sEVs significantly increased the proportion of Ki-67 positive cells after 72 h (p = 0.03572, n = 4; Fig. 5b,c).

Proliferation of MSCs was also tested by direct cell counting, where all of the cells in the sEV-exposed cell cultures were counted using DAPI staining, imaging and machine learning. Results showed increased proliferation of cells upon exposure to Hs and Ag-TiO_2_ sEVs as early as 24 h, but different sEVs each had a distinct influence on this cell function (Fig. 5d).

Our *in vitro* results suggest that melanoma sEVs released under different microenvironmental conditions may have distinct effect on stem cell proliferation. However, beside the IPA predicted interactions, additional molecules and factors, such as the encapsulated doxorubicin, may also be involved in this process.

Previously, tumour exosomes derived from melanoma cells^17^, osteosarcoma cells^63^ or breast cancer cells^64^ have been shown to re-educate MSCs and provide them tumour-promoting properties. The tumour-educated stem cells may go through an oncogenic reprogramming resulting in increased proliferation capacity *in vitro*, and tumour growth- and metastasis progression-supporting effects *in vivo*. Here, we also observed an increase in the proliferation and Ki-67 expression upon the normal (Ctrl) sEV exposure. However, our results suggest that the stem cell re-education capacity of vesicles strongly depends on the microenvironmental conditions of the releasing tumour cells.

#### Doxo and Ctrl sEVs affect the cell cycle of melanoma cells

**Figure 6:**
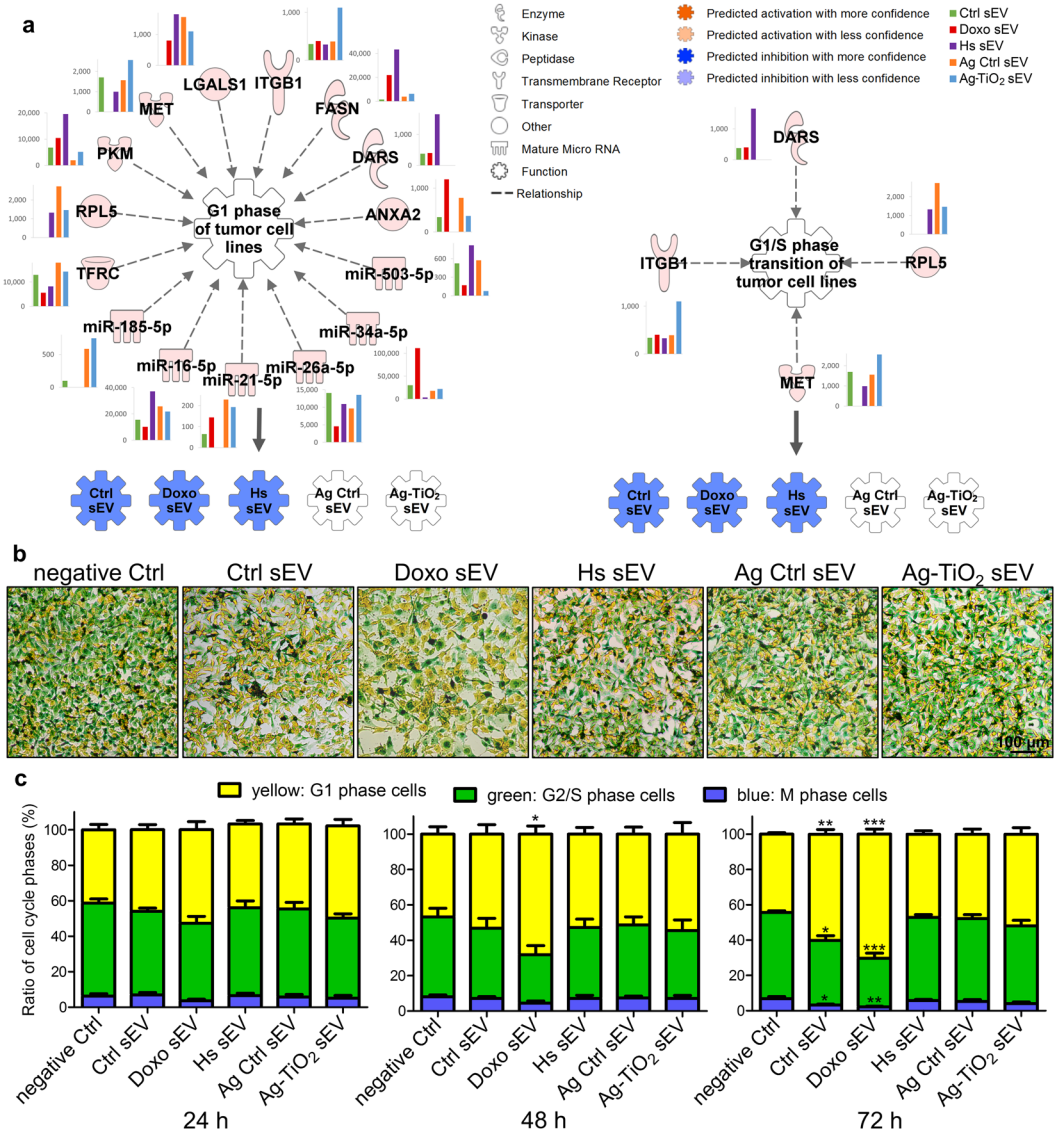
Ctrl and Doxo sEVs caused an arrest in G1 phase of melanoma cells. (**a**) IPA predictions for the regulatory effects of sEV molecules on the ‘G1 phase of tumour cell lines’ and ‘G1/S phase transition of tumour cell lines’. Networks show every upstream regulator proteins and miRNAs accompanied by a bar graph, which represents the normalised expression values of the molecule for each sEV group. Coloured symbols, named as the sEV groups, display the expected regulation changes of the analysed ‘Biofunctions’ upon exposure to the vesicles. (**b**,**c**) Cell-Clock cell cycle assay of sEV-exposed B16F1 cell cultures. (**b**) Representative images of the cell clock dye-labelled cultures. (**c**) Distribution of the yellow, green and blue cells in the cell cultures, which labels the G1, G2/S and M phase cells, respectively. Each bar represents mean + SD (n = 4), *p < 0.05, **p < 0.01 and ***p < 0.001 indicate statistical significance.

IPA analyses predicted inhibition of ‘G1 phase of tumour cell lines’ and ‘G1/S phase transition of tumour cell lines’ upon exposure to Ctrl, Doxo and Hs sEVs (Fig. 6a). In other words, the molecular content of these vesicles may cause an arrest in the G1 phase in recipient tumour cells. As displayed on Fig. 6a, IPA found a total of 15 sEV molecules in our B16F1 data, which may influence the G1 phase of tumour cell lines. Hypothetically, the key player of their inhibitory effect may be the aspartyl-tRNA synthetase (DARS). In 1999, Yamashita *et al*.^65^ investigated T24 bladder carcinoma cells and showed that DARS causes a retinoblastoma-independent downregulation of cyclin A, which is required for S phase entry^65^. Another component of the B16F1 vesicles, the p53 inducible miR-34a may lead to apoptosis and cell cycle arrest in the G1 phase, thereby suppressing tumour cell proliferation^66^. Ji *et al*.^67^ showed that restoration of miR-34 expression in pancreatic cancer cells inhibited cell growth and invasion, induced apoptosis, arrested cell cycle in G1 and G2/M phases and sensitized the cells to chemotherapy and radiation^67^.

To test the *in silico* predicted effects of sEVs on tumour cell cycle, we performed a Cell-Clock cell cycle assay on B16F1 melanoma cells exposed to sEVs for 24 h, 48 h and 72 h along with a PBS-treated negative control group. This assay utilizes a vital redox dye that changes colour based on the cell cycle phase. It becomes yellow in G1, green in S/G2 and blue in M phase. In the negative Ctrl group, cells were present in the G1, S/G2, and M phases in average proportions of 44.84 ± 2.68%, 47.80 ± 3.34% and 7.35 ± 0.81%, respectively (n = 12). Ctrl and Doxo sEVs led to an increase in the proportion of the yellow, *i.e*. G1 phase, cells in a time dependent manner. After 72 h, these cells represented 59.20 ± 4.06% of the Ctrl sEV-exposed cultures (p = 0.00346, n = 4) and 70.32 ± 7.24% of Doxo sEV-exposed cultures (p = 4.28 × 10^−6^, n = 4) (Fig. 6b,c). These results confirmed the IPA predicted arrest in G1 phase by the Ctrl and Doxo, but not by the Hs sEVs.

It has been previously demonstrated that tumour cells can efflux drugs through exosome secretion^43,68^. Yang *et al*. showed that doxorubicin-treated MCF-7 breast carcinoma cells produced drug-containing exosomes. Doxorubicin that was encapsulated in the MCF-7 exosomes had more potent cytotoxicity against the parental MCF-7 cells than the free drug^43^. Furthermore, doxorubicin arrest the cell cycle of tumour cells at G1/S and G2/M checkpoints^69,70^. Based on these literature data and our fluorescence spectroscopy measurements, we hypothesize that encapsulated doxorubicin might contribute to the enhanced cell cycle arrest effect of Doxo sEVs.

#### Migration capacity of melanoma cells is differently altered by the sEVs

**Figure 7:**
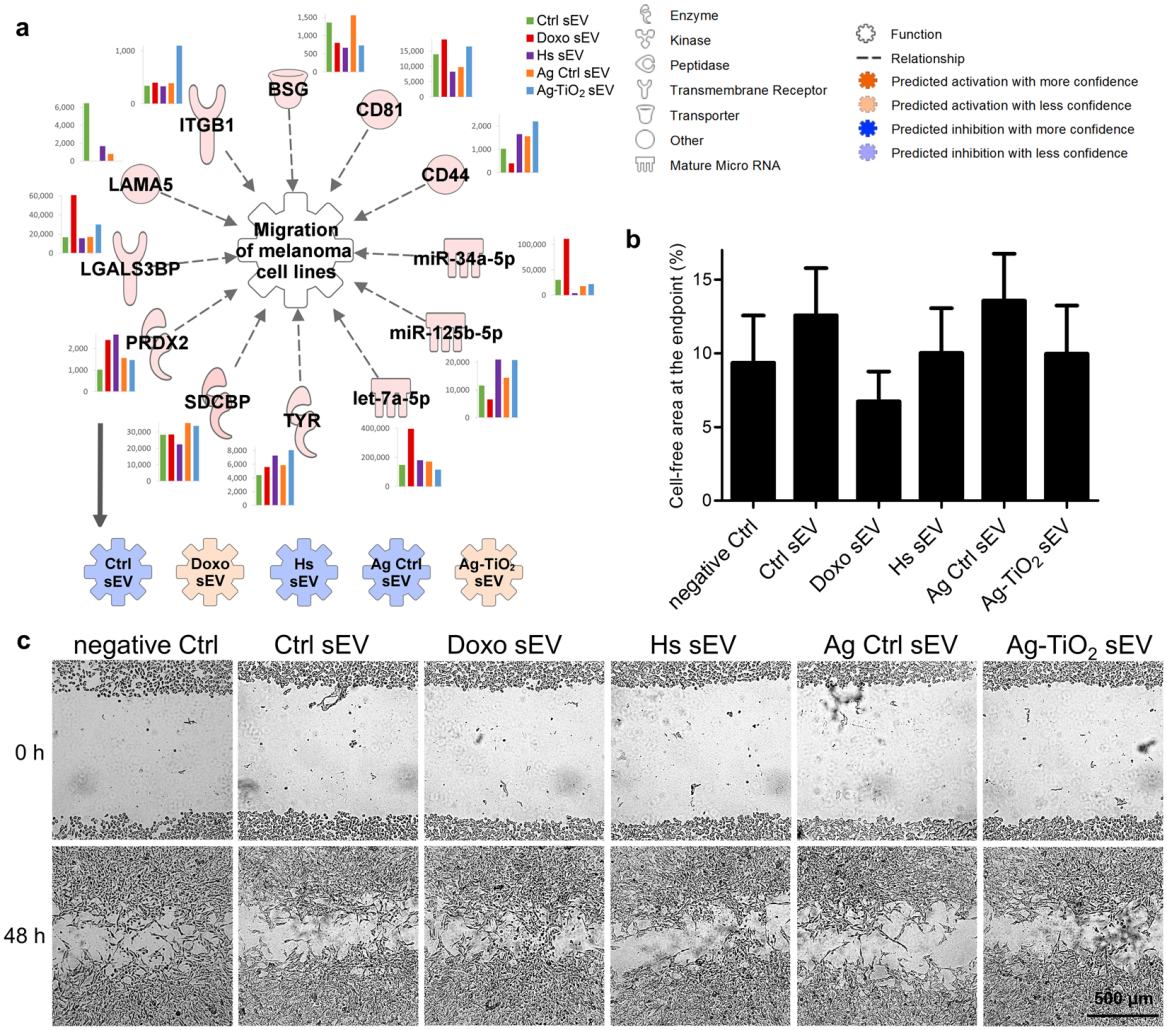
Doxo sEVs enhanced the migration of melanoma cells. (**a**) IPA predictions for the regulatory effects of sEV molecules on the ‘Migration of melanoma cell lines’. Network shows every upstream regulator proteins and miRNAs accompanied by a bar graph, which represents the normalised expression values of the molecule for each sEV group. Coloured symbols, named as the sEV groups, display the expected regulation changes of the analysed ‘Biofunction’ upon exposure to the vesicles. (**b**,**c**) Wound healing assay of sEV-exposed B16F1 cell cultures. (**b**) The bar graph shows the result of the analysis of wound closures by the ImageJ wound healing tool. It represents mean + SD values (n = 8). (**c**) Representative images of the wounds after 48 h of sEV exposures.

From many cell movement-related ‘Biofunctions’, which were predicted to be targeted by sEVs (Fig. 4d–f), we chose the ‘Migration of melanoma cell lines’ for further IPA and *in vitro* investigations. The *in silico* analyses showed varying sEV effects. More specifically, Doxo and Ag-TiO_2_ sEVs are predicted to facilitate the melanoma cell migration, while the three other ones may inhibit this function (Fig. 7a). According to the IPA analyses, negative B16F1 sEV regulators may include the laminin subunit alpha-5 (LAMA5), peroxiredoxin-2 (PRDX2), tyrosinase (TYR), let-7a-5p, miR-125b-5p and the miR-34a-5p. Listed positive regulators include the CD44 antigen (CD44), CD81 antigen (CD81), basigin (BSG), integrin beta-1 (ITGB1) and the galectin-3-binding protein (LGALS3BP) (Fig. 7a). Using attachment and pulmonary metastases assays, Hibino *et al*.^71^ identified four peptides of LAMA5, which showed activity *in vitro* and also *in vivo*. These peptides reduced migration and invasion of B16F10 melanoma cells^71^. Lee *et al*.^72^ showed that the PRDX2 enzyme is a selective antioxidant suppressor for proliferation and migration of melanoma cell lines (SK-MEL-5, SK-MEL-28, A375, G361, B16F10)^72^. Stampolidis *et al*. demonstrated that LGALS3BP promotes cell viability and facilitates cell motility of the human C8161 melanoma cell line^73^.

Using wound healing assay, we investigated the effect of sEVs on migration of B16F1 cells, which approximated to the IPA predicted tendency. Migration of cells into the wounded area was slightly decreased in the presence of Ctrl and Ag Ctrl sEVs, compared to migration of the negative Ctrl cells. Acceleration of wound closure was observed in response to Doxo sEVs (n = 8). However, Hs and Ag-TiO_2_ sEVs had no effect on tumour cell migration (Fig. 7b,c).

The importance of migration capacity in tumour progression is unquestionable, since the process of tumour cell invasion and metastasis is conventionally understood as the migration of individual cells, which detach from the primary tumour, enter lymphatic vessels or the bloodstream and seed in distant organs^74^. This cancer cell migration is typically regulated by integrins, matrix-degrading enzymes, cell-cell adhesion molecules and cell-cell communication^74^. Direct and indirect effects of stress-elicited sEVs on tumour cell migration and metastasis have been demonstrated in some studies^27,47,75^. Here, we amended literature data by showing that the cytostatic stress-exposed cell-derived sEVs enhance the migration of the recipient melanoma cells. This can be interpreted as an adaptive escape mechanism: Doxo sEVs, delivering a warning message, induce the migration of the neighbouring melanoma cells. In a recent study^27^, breast cancer cells also showed an sEV-mediated escape mechanism under doxorubicin and paclitaxel exposures, by releasing pro-metastatic exosomes^27^.

#### Each sEV group enhances the migration of endothelial cells

IPA predicted the activation of ‘Cell migration of endothelial cells’ and ‘Cell spreading of endothelial cells’ upon exposure to each sEV group labelling a large number of potentially contributing sEV molecules (Supplementary Fig. S5). Therefore, we repeated the wound healing assays on bEnd.3 mouse endothelial cells, where we could verify the IPA predictions. However, Ctrl sEVs showed the highest migration enhancing effect (Supplementary Fig. S5).

Endothelial cell migration is an essential component of angiogenesis, which is a key process of tumour progression^76^. These results demonstrate that some type of stress conditions may slightly decrease the endothelial cell migration promoting effects of sEVs. At the same time, they highlight that sEVs may have target cell-specific functional effects in the recipients, which further increases the diversity of the sEV-mediated communication of melanoma cells.

#### Microtissue generation is facilitated independently of the sEV groups

**Figure 8:**
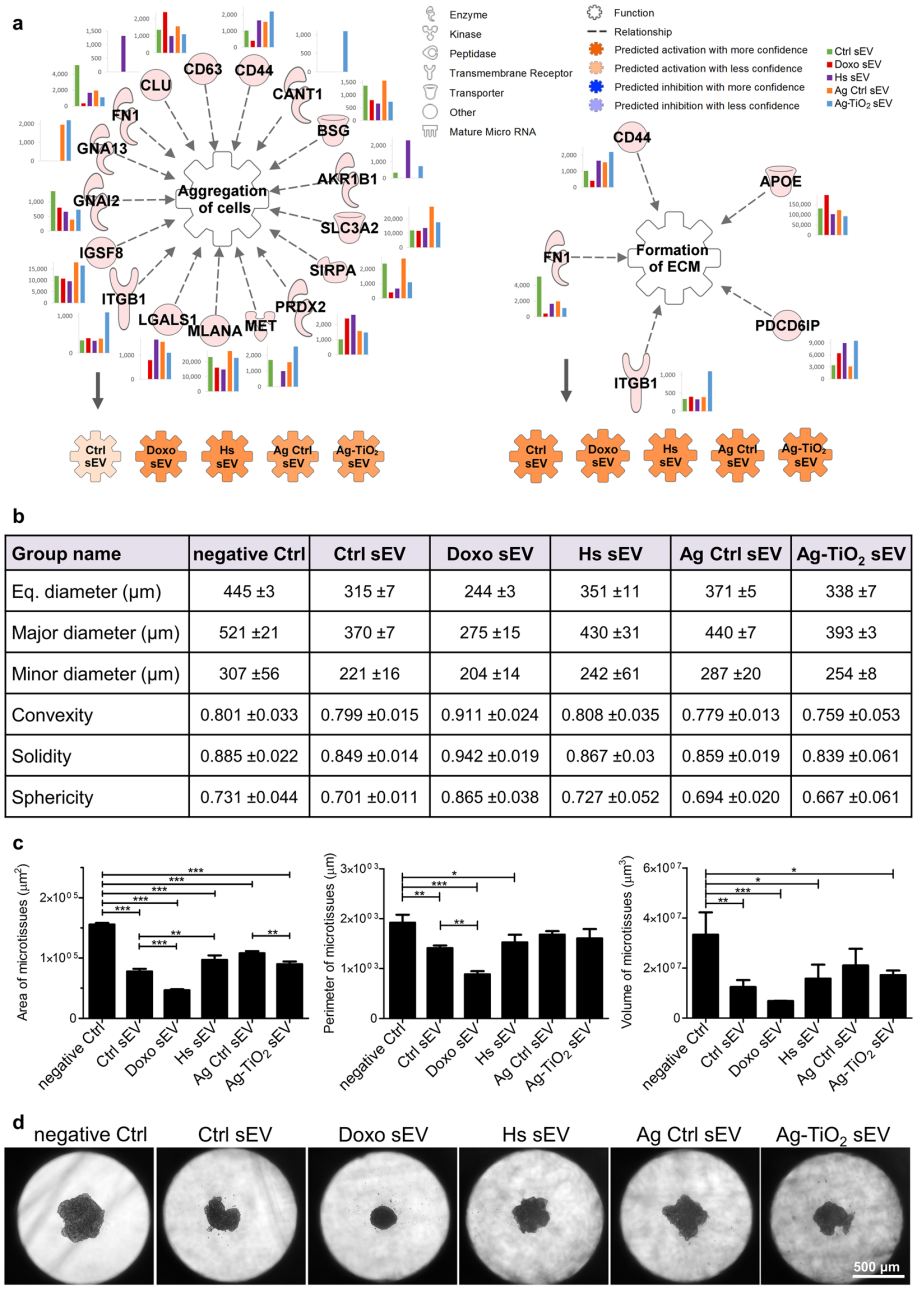
All sEV groups facilitated microtissue generation. (**a**) IPA predictions for the regulatory effects of sEV molecules on the ‘Aggregation of cells’ and the ‘Formation of extracellular matrix’. Networks show every upstream regulator proteins and miRNAs accompanied by a bar graph, which represents the normalised expression values of the molecule for each sEV group. Coloured symbols, named as the sEV groups, display the expected regulation changes of the analysed ‘Biofunctions’ upon exposure to the vesicles. (**b**,**c**) Descriptive statistics of the 72 h B16F1-MSC microtissues resulted from image analysis using the AnaSP software. Eq. diameter means equivalent diameter, major and minor diameters are measured through centroid. Table contains mean ± SD values. Bar graphs show the area, perimeter and volume statistics of the generated microtissues (mean + SD, n = 3). Statistical evaluation was performed by Welch’s ANOVA test with Tukey’s HSD post-hoc test; *p < 0.05, **p < 0.01 and ***p < 0.001 indicate statistical significance. (d) Representative images of the generated microtissues after 72 h of sEV exposures.

IPA predicted that each of the five sEV groups may activate many ‘Biofunctions’ related to the formation of a 3D cell interaction matrix, *e.g*. ‘Aggregation of cells’, ‘Formation of ECM’ (Fig. 8a), ‘Cell-cell contact’ or ‘Interaction of tumour cell lines’ (Supplementary Fig. S6). The intensity of these activations is variable between sEV groups, for example activation of the ‘Aggregation of cells’ is predicted to be the strongest upon Doxo and Hs sEV exposures. *In silico* analyses revealed a large number of contributing B16F1 sEV molecules. For instance, it is worth to mention the programmed cell death 6-interacting protein (PDCD6IP, Alix). It has an important role not only in the exosome biogenesis^52^, but Pan *et al*.^77^, who investigated fibroblast morphology, demonstrated that a sub-population of Alix localises extracellularly and regulates integrin-mediated cell adhesions and fibronectin matrix assembly^77^.

In order to mimic *in vivo* conditions, we established a simplified 3D tumour matrix model co-culturing MSCs and B16F1 cells in hanging drop plates. By definition, multicellular cell aggregates, which produce their own ECM and are comprised of more than one cell types are termed as microtissues^78^. Applying an equal number of the two cell types (a total of 5,000 cells/well), microtissue generation was followed under sEV exposures and PBS treatment as a negative control for 72 h. The created microtissues were photographed and analysed by the AnaSP software developed for automatic image analysis of multicellular spheroids^79^. Each group of sEVs resulted in smaller and more compact microtissues, since the average values of the measured area, perimeter, diameter and volume was lower, compared to the negative Ctrl samples (Fig. 8b–d). These results were validated using mouse embryonic fibroblasts (MEFs) co-cultured with B16F1 cells, where each sEV group facilitated the microtissue generation, except the Hs sEVs (Supplementary Fig. S7).

In videomicroscopy studies, Crawford *et al*.^80^ observed that microvesicles attaching to the cell membranes promoted cell-to-cell interactions and spheroid formation of glioblastoma, breast carcinoma and osteosarcoma cells. They also showed that elevated extracellular Ca^2+^ levels promote microvesicle production and result in smaller and less dense spheroids, which might seem paradoxical. However, they interpreted their results as a pathophysiological adaptation, since increasing spheroid surface to volume ratio, thereby increasing the surface area enhances the uptake of oxygen, growth factors and nutrients by tumour cells that make up the spheroid mass^80^. In our experimental setup, sEV exposures resulted in smaller microtissues from the same number of cells suggesting the generation of more compact structures, which may be explained by the role of vesicles in generation of cell-cell associations, and may also indicate an adaptation mechanism.

Tumours exist in a 3D microenvironment, in which morphological and functional properties, such as the ECM, cell-cell interactions, oxygen gradient and acid gradient create transport barriers for drug delivery^81^. In this study, Doxo sEV exposure resulted in the most compact structures, which may reduce penetration of drugs. Previously, tumour-derived extracellular vesicles have been shown to transfer drug resistance into other tumour cells^82,83^ suggesting that the Doxo sEVs might convey protective messages resulting in more compact microtissues.

## Conclusions

In this study, we compared different inducible cellular stress conditions, and we found that cytostatic, heat and oxidative stresses resulted in changes in the vesicular cargo composition, leading to distinct functional alterations of the melanoma-derived sEVs in the TME recipient cells.

We showed that sEVs, being complex information packages may participate in a wide range of signalling pathways. The fact that a vesicular molecular pattern with a large number of molecules can influence the activation of the cellular homeostasis network at several points, suggests a huge diversity of sEV functions, which sometimes seem to be paradoxical in the *in vitro* experiments. In conclusion, hundreds of vesicular molecules may have thousands of functional effects in the recipient cells leading to an unconceivable outcome. Here, we successfully predicted the functional effects of the investigated sEV molecular patterns – induced by five treatment conditions – by bioinformatics analyses using unique combinations of the IPA approaches. Based on our knowledge, our experimental setup was suitable to model the transfer and functional activity of the vesicular cargo in the recipient cells. Here, we demonstrated, that pathway analyses may provide a good approximation to the prediction of different inducible stress-elicited responses, suggesting that *in silico* analyses may be useful tools not only in the field of research, but in a therapeutic approach as well.

Our paper not only provides a detailed characterisation of the doxorubicin-, heat- and Ag-TiO_2_-induced molecular patterns of melanoma-derived sEVs and the resulted response patterns in the recipient cells, it also increases our knowledge about the molecular and functional complexity as well as condition-dependent variability of the melanoma-derived sEVs. This study contributes to a better understanding of the pathogenesis and therapeutic responses of melanoma. It also highlights that indirect effects of any therapy, such as a chemotherapy, may have a great influence on the intercellular communication of the affected cells.

Based on this study, we conclude that the molecular pattern of these highly protected information packages is dictated by the microenvironmental conditions, including the therapeutic stress factors. The altered cargo of sEVs is able to not only enhance or suppress existing signalisation pathways, but even trigger *de novo* pathway activations, resulting a unique target cell-specific response pattern in the sEV recipient cells. Recent literature data^24,25,27,47^ along with this study suggest that alteration of this complex sEV-mediated intercellular communication of tumour cells deserves special attention among the therapy-induced host responses, which may have a potential influence on the treatment efficacy.

## Methods

### Cell cultures

B16F1 (ECACC 92101203) mouse melanoma cell line was obtained from ECACC and cultured in DMEM supplemented by 10% FBS (EuroClone), 2 mM L-glutamine and 1% Penicillin-Streptomycin-Amphotericin B mixture (P/S/A; all from Lonza); mouse embryonic fibroblasts (MEFs; ATCC SCRC-1040) were obtained from ATCC and cultured in DMEM supplemented by 15% FBS and 1% P/S/A; bEnd.3 mouse endothelial cell line (ATCC CRL-2299) was obtained from ATCC and cultured in DMEM supplemented by 10% FBS and 1% P/S/A. Primary mouse mesenchymal stem cells (MSCs) were isolated from adipose tissue of 6-8 week old male C57BL/6N mice (Charles River Laboratories) and cultured using the MesenCult Expansion Kit (STEMCELL Technologies). All cell cultures were maintained in a humidified incubator at 37°C and 5% CO_2_. Purity of MSC cultures was checked by flow cytometry using the Mouse Multipotent Mesenchymal Stromal Cell Marker Antibody Panel (R&D Systems) according to the manufacturer’s instructions and a FACSCalibur instrument coupled with CellQuest Pro 6.0 (BD Biosciences). Animal experiments were performed in accordance with the national and European animal ethics guidelines. The animal experimental protocols were approved by the Animal Experimentation and Ethics Committee of the Biological Research Centre of the Hungarian Academy of Sciences and the Hungarian National Animal Experimentation and Ethics Board (clearance number: XVI./78/2018).

### Ag-TiO_2_ photocatalyst particles

Synthesis of Ag-TiO_2_ nanoparticles was performed as published earlier^31,35^. Briefly, the silver particles were strongly attached on the surface of metal oxide by photodeposition. A commercially available TiO_2_ (Degussa P25, Evonik Gmbh) with a specific surface area of ~50 m^2^/g was used for this purpose. The prepared Ag-TiO_2_ photocatalyst contained 0.5 wt% surface silver nanoparticles. Dispersion of particles was made in PBS at a concentration of 10 mg/ml and sonicated for 30 min directly before use.

### Stress conditions

B16F1 cell cultures were treated at 70% confluency in five different ways in EV-depleted FBS-containing media for 72 h (Table 1). The Control 1 group received only fresh medium. Cytostatic stressed cells were treated with 0.6 μM doxorubicin, heat stressed cells were incubated at 42°C for 2 h in every 24 h (a total of 3 times), oxidative stressed cells were treated with light-induced 2.5 μg/ml Ag-TiO_2_. In detail, the photoreactive Ag-TiO_2_ nanoparticles were induced by a low-pressure mercury lamp (λ ≥ 360 nm, GCL303T5/4 type, LightTech) for 60 min from 3 cm distance in a 75% medium volume to avoid the light absorption and reflection of the medium layer. After the illumination, the medium was adjusted to the final volume. To eliminate the effect of illumination itself, we established another control group, which received medium illuminated in the same way as described above. Parameters of the cytostatic and oxidative stresses were based on previous optimization by proliferation assay. Heat stress conditions were adapted from literature data^34^. In each group, 72 h supernatants of 6 parallel cell cultures were harvested, pooled and subjected to sEV isolation.

### Scanning electron microscopy (SEM)

B16F1 cells seeded to poly-L-lysine-coated 5 mm cover glasses were treated as described above. After 24 h incubation, cells were washed with PBS and fixed for overnight in 2.5% glutaraldehyde and 0.05 M cacodylate buffer diluted in PBS (pH 7.2). Then, cells were washed with PBS, dehydrated with a graded ethanol series (30%, 50%, 70%, 80% EtOH, each for 1 h and 100% EtOH, for 3 × 1 h) and dried with a critical point dryer (Quorum Technologies Ltd, K850). Cover glasses were mounted onto microscope stubs using carbon tape, followed by 15 nm gold coating (Quorum Technologies Ltd, Q150) and observed under a field-emission scanning electron microscope (JEOL Ltd, JSM-7100F/LV). Images were taken in 1,500 × and 20,000 × magnification.

### sEV isolation and characterisation

Vesicles were isolated by differential filtration and ultracentrifugation. Briefly, 72 h supernatants from the stress-exposed B16F1 cultures were centrifuged at 780 g for 5 min, and at 3,900 g for 15 min at 4°C, then filtered by a 0.2 µm membrane to remove cells, debris and larger vesicles. Small EVs were pelleted by ultracentrifugation at 150,000 g for 60 min at 4°C using a T-1270 fixed-angle rotor and a WX + ultracentrifuge (Sorvall). The pellet was washed twice and resuspended in PBS. Protein concentrations of sEV isolates were measured by the Pierce BCA Protein assay kit (Thermo Scientific) on a benchtop microplate reader (Multiskan RC, Thermo Labsystems) coupled with the Ascent Software 2.6. Small EVs were characterised by atomic force microscopy as described previously^44^, dynamic light scattering using a Zetasizer Nano S instrument (Malvern Panalytical Ltd) and Western blot analysis (described in the Supplementary Methods). Quantification of sEVs was performed by nanoparticle tracking analysis using a NanoSight NS500 instrument (Malvern Panalytical Ltd).

### Fluorescence spectroscopy

The emission and excitation spectra of doxorubicin were measured by a FluoroLog-3 spectrofluorometer (Horiba Ltd). The maximum wavelengths for excitation (λ_ex_ = 492 nm) and emission (λ_em_ = 592 nm) were then used for measuring the fluorescence intensity of Doxo sEVs and Ctrl sEVs as a background. A calibration curve of doxorubicin covering a concentration range of 0-1,000 nM was applied to determine the encapsulated doxorubicin concentration of the sEV samples.

### Dynamic light scattering (DLS) measurements of Ag-TiO_2_ particles

The particle size values of the Ag-TiO_2_ photocatalyst particles were determined DLS with a Zetasizer Nano ZS ZEN 4003 apparatus (Malvern Panalytical Ltd) equipped with a He-Ne laser (λ = 633 nm). The measurements were performed in B16F1 culture medium for a 72 h time interval. Size distribution measurements were carried out in triplicate, and mean ± SD values are reported.

### Chemiluminescence detection of Ag-TiO_2_ nanoparticles

Isolated Ag Ctrl and Ag-TiO_2_ sEVs were lysed by TENT buffer and freeze-thaw cycle, then a 10-fold, 5-step Ag-TiO_2_ nanoparticle dilution series (0–2.5 µg/ml) was prepared in Ag Ctrl sEV suspension for calibration. Protein concentration of sEV lysates was 200 µg/ml. After 30 min illumination on 96-well plates, 50 µl 3.38 mM luminol solution was added to 50 µl of samples and light emission was immediately detected by a Luminoskan Ascent Microplate Luminometer (Thermo Scientific). Each sample was measured in triplicates.

### Transmission electron microscopy (TEM)

The morphology of Ag-TiO_2_ sEVs and Ag-TiO_2_ sEVs mixed with Ag-TiO_2_ nanoparticles was examined using a Tecnai G2 20 × -Twin type instrument (FEI), operating at an acceleration voltage of 200 kV. For TEM measurements the samples were dropped on a grid (carbon film with 200 Mesh coper grids; CF200-Cu, Electron Microscopy Sciences) and dried.

### miRNA analysis of sEVs

Pellets of sEVs were subjected to miRNA isolation using the NucleoSpin miRNA isolation kit (Macherey-Nagel) according to the manufacturer’s instructions. Sequencing was performed using SOLiD Total RNA-Seq lit for Small RNA Libraries (Applied Biosystems) based on the manufacturer’s protocol. Purification was performed on 10% TBE-Urea gels stained with Sybr Gold nucleic acid gel stain (both from Invitrogen). Final purification was performed using PureLink PCR Micro Kit (Invitrogen). Final libraries were quality checked using High Sensitivity DNA kit on Bioanalyzer (Agilent Technologies). Concentration of the libraries was determined by the SOLiD Library TaqMan Quantitation Kit (Life Technologies). Each library was clonally amplified on SOLiD P1 DNA Beads by emulsion PCR (ePCR). Emulsions were broken using butanol, and ePCR beads were enriched for template-positive beads by hybridization with magnetic enrichment beads. Template-enriched beads were extended at the 3’ end in the presence of terminal transferase and 3’ bead linker. Beads with the clonally amplified DNA were deposited onto SOLiD sequencing slide and sequenced on SOLiD 5500xl instrument using the 50-base sequencing chemistry.

Bioinformatics analysis of raw data, quality assessment, read trimming, read mapping and miRNA expression profiling was carried out in CLC Genomics Workbench 8.0.2 (Qiagen Bioinformatics) using annotated *Mus musculus* miRNA sequences according to the miRBase release 21 as a mapping reference. Only miRNAs with ≥10 read counts were accepted.

Results of sequencing were validated by qPCR on 3 selected miRNAs, mmu-miR-16-5p, mmu-miR-125b-5p, mmu-miR-29a-3p. Intact total RNA – including miRNA – were prepared from sEV isolates by miRNA Miniprep System (Promega) according to the manufacturer’s instructions. Then, 70 ng of each sample were reverse transcribed using microRNA cDNA synthesis kit (Sigma-Aldrich). The qPCR reactions were performed on PikoReal Real-Time PCR System (Thermo Scientific) using SYBR Green chemistry and commercially available miRNA specific primers (Sigma-Aldrich). Cq values of each miRNA were normalized by U6 endogenous controls in all samples and expression levels were calculated using -ddCt method.

### Proteome analysis of sEVs

Detailed LC-MS/MS analysis of sEVs is described in the Supplementary Methods. Briefly, 25 µg of vesicular proteins were separated by SDS-PAGE and stained with Coomassie blue. Then, each lane was cut to 12 equal bands and subjected to an in-gel trypsinisation procedure. The extracted peptides were analysed on an LTQ-Orbitrap Elite (Thermo Scientific) mass spectrometer on-line coupled with a nanoHPLC (nanoAcquity, Waters) system. Searchable peaklists were extracted using Proteome Discoverer 1.4 (Thermo Scientific) and subjected to database search on our in-house Protein Prospector 5.14.1 search engine against the *Mus musculus* and *Bos taurus* protein sequences of the Uniprot (UniProtKB.06.11.2014) database completed with human keratins and pig trypsin, altogether 106,330 protein sequences were searched. Protein identification was accepted if the protein was identified with ≥3 unique peptides, but peptides with identical bovine and mouse sequence were excluded. FDR values were less than 1% in all cases. Results were validated by Western blot (described in the Supplementary Methods).

### Bioinformatics analysis

Normalised miRNA and protein data derived from the LC-MS/MS and SOLiD sequencing were analysed by the Ingenuity Pathway Analysis (IPA, Qiagen Bioinformatics). First, we used the ‘Core Analysis’ feature to reveal functional differences between the five sEV groups, where ‘Top 5 canonical pathways’ and ‘Top 5 molecular and cellular functions’ (Fig. 4a,b) were obtained. Secondly, using the ‘Comparison Analysis’ feature, we created a heatmap containing ‘Biofunctions’, which had relevance in melanoma and >5 -log(p-value). This heatmap was divided into four parts based on biological relevance; intracellular, cellular, systemic and immune processes are displayed in separated panels (Fig. 4c–f). Thirdly, some ‘Biofunctions’ were chosen for further investigation to reveal the regulatory effects of sEVs on them. Using the ‘Grow tool’, the upstream interacting vesicular molecules were identified for the selected ‘Biofunctions’ for each sEV group. Then, using the ‘Molecule Activity Predictor’ (MAP) tool, we could reveal the activation or inhibitory effects of each sEV group for each ‘Biofunction’ (Figs. 5–8a). Through these *in silico* analysis, we could model the effects of the different sEVs in the recipient cells in spite of their molecular complexity. Figures were edited in the IPA ‘Path Designer’ and completed with Excel diagrams. For all IPA analyses, the confidence level was set to ‘Experimentally observed’ enabling literature data-based analysis, but not unproven predictions.

### Exposures of MSCs and B16F1 cells to the sEVs

To avoid additional effects of changing conditions, cells were exposed to sEVs in their standard, complete media before each of the following functional assays. Briefly, cells were treated with 200 µg/ml sEV suspensions, or PBS as a negative control for 24 h, 48 h or 72 h. For longer incubation times, treatments were repeated in every 24 h.

### Ki-67 expression analysis of MSCs

MSCs exposed to sEVs for 24 h and 72 h were fixed in 4% paraformaldehyde for 10 min at room temperature (RT) for immunocytochemistry. Then, cells were permeabilised with 0.1% Triton X-100 and non-specific antibody binding was blocked with 5% BSA. We applied direct labelling using anti-mouse/rat Ki-67 monoclonal antibody conjugated to eFluor 615 dye (1:400, eBioScience) in 1.2% BSA overnight at 4°C. Nuclear counterstaining was performed with DAPI for 15 min at RT. Cells were washed 3 times with PBS for 5 min between each step. Finally, the cells were covered by Fluoromount-G (SouthernBiotech) and cover glasses. Fluorescent images were taken by the Operetta high content screening system (PerkinElmer) and analysed by an image analysis and machine learning software (SCT Analyzer 1.0) developed by the Single-Cell Technologies Ltd^62^. Our pipeline was comprised of cell segmentation, feature extraction and machine learning modules. K-means algorithm was used for the nuclei segmentation based on the DAPI signal, then we extracted the eFluor 615 signal-related features, *i.e*. max intensity, min intensity, mean intensity, median intensity, SD intensity for the generated nuclei masks. We established a training set with two classes for the Ki-67 positive and the negative cells. This training set, containing 100 objects in both classes was validated by the implemented k-fold cross-validation. For machine learning, we used the Multi-Layer Perceptron (MLP) method. The Ki-67 expression analysis was repeated 4 times and the applied methods enabled to analyse a total of 159,596 cells.

### Cell counting

MSC cultures in 384-well plates were exposed to sEVs for 24 h and 72 h, then fixed in 4% paraformaldehyde for 10 min at RT and stained with 1 µg/ml DAPI for 15 min at RT. Images were acquired from whole wells using a TCS SP8 microscope (Leica Microsystems) in fluorescent mode, followed by an analysis using the SCT Analyzer 1.0 machine learning software. The experiment was repeated 3 times.

### Cell cycle analysis

Changes in the cell cycle dynamics of sEV-exposed B16F1 cells were analysed using the Cell-Clock cell cycle assay (Biocolor Ltd) according to the assay protocol. This assay can be used to distinguish the four major phases of the mammalian cell cycle using a vital redox dye, which is yellow, green or dark blue in G1, S/G2, and M phase cells, respectively. After staining, cells were photographed using an Axiovert S100 microscope (Zeiss) equipped by a Nikon D5000 camera. Images were analysed by the ImageJ software to determine the percentage of cells in each cell cycle phase. The experiment was performed with 4 repeats.

### Wound healing assay

Alterations of the migration capabilities of sEV-exposed B16F1 and bEnd.3 cells were assessed by scratch assay. Nearly confluent monolayers of cells were scratch wounded using a sterile 200 µl pipette tip, washed 3 times with culture media to remove cellular debris, then treated with 200 µg/ml sEV suspensions or PBS in fresh complete media. Wound closure was followed until the cell-free area decreased below 10% in at least 1 sample, when images were taken by an inverted microscope (Zeiss, Axiovert S100) equipped by a Nikon D5000 camera. Grey-scaled images were analysed using the MRI Wound Healing Tool in the ImageJ software. The experiment was repeated 8 times for B16F1 cells, and 4 times for bEnd.3 cells.

### Analysis of microtissue generation

Effects of different sEVs on cell-cell contact and cell-ECM interactions was examined on MSC-B16F1 and MEF-B16F1 co-cultures using a simplified 3D tumour matrix model to better represent the *in vivo* conditions, than 2D cultures. Equal number of MSCs or MEFs and B16F1 cells were seeded to 96-well GravityPLUS hanging drop plates (InSphero AG) in sEV- or PBS-containing media (5,000 cell/40 µl/well). Microtissue generation was followed for 72 h and images were acquired in every 24 h using an Axiovert S100 microscope (Zeiss) equipped by a Nikon D5000 camera. To quantify differences in size and shape between the microtissues, 72 h images were grey scaled and analysed by the AnaSP software^79^. Measured parameters of microtissues were the equivalent diameter, major diameter through centroid, minor diameter through centroid, convexity, solidity, sphericity, area, perimeter and volume. The experiments were repeated 3 times.

### Statistical analysis

Since the homogeneity of variances assumption of the ANOVA had not met with our data, statistical analyses were performed by the Welch’s ANOVA test with Tukey’s HSD post-hoc test (Alpha = 0.05) using a Microsoft Excel add-in, the Real Statistics Resource Pack software (Release 5.4). Copyright (2013–2018) Charles Zaiontz (www.real-statistics.com). Diagrams were prepared in GraphPad Prism 5.03. All average values represent mean ± SD and number of asterisk denote minimum statistical significance, *i.e*. *p < 0.05, **p < 0.01 and ***p < 0.001 on figures. Exact p-values are indicated in the text, when it is necessary. Figure 1 was created with BioRender.com.

## Supplementary information

Supplementary information

## Data Availability

All datasets generated during the current study are available from the corresponding author upon reasonable request.

## References

[1] Kotrbová A (2019). TEM Exosome Analyzer: a computer-assisted software tool for quantitative evaluation of extracellular vesicles in transmission electron microscopy images. J Extracell Vesicles..

[2] Yáñez-Mó M (2015). Biological properties of extracellular vesicles and their physiological functions. J Extracell Vesicles..

[3] Kolenda T (2018). Tumor microenvironment - Unknown niche with powerful therapeutic potential. Rep Pract Oncol Radiother..

[4] Meehan K, Vella LJ (2016). The contribution of tumour-derived exosomes to the hallmarks of cancer. Crit Rev Clin Lab Sci..

[5] Javeed N, Mukhopadhyay D (2017). Exosomes and their role in the micro-/macro-environment: a comprehensive review. J Biomed Res..

[6] Tkach M, Théry C (2016). Communication by Extracellular Vesicles: Where We Are and Where We Need to Go. Cell..

[7] Whiteside TL (2016). Tumor-Derived Exosomes and Their Role in Cancer Progression. Adv Clin Chem..

[8] Braeuer RR (2014). Why is melanoma so metastatic?. Pigment Cell Melanoma Res..

[9] Matsumoto A (2017). Accelerated growth of B16BL6 tumor in mice through efficient uptake of their own exosomes by B16BL6 cells. Cancer Sci..

[10] Guo D (2019). RAB27A promotes melanoma cell invasion and metastasis via regulation of pro-invasive exosomes. Int J Cancer..

[11] Li J (2019). Blockage of transferred exosome-shuttled miR-494 inhibits melanoma growth and metastasis. J Cell Physiol..

[12] Isola AL, Eddy K, Zembrzuski K, Goydos JS, Chen S (2017). Exosomes released by metabotropic glutamate receptor 1 (GRM1) expressing melanoma cells increase cell migration and invasiveness. Oncotarget..

[13] Xiao D (2016). Melanoma cell-derived exosomes promote epithelial-mesenchymal transition in primary melanocytes through paracrine/autocrine signaling in the tumor microenvironment. Cancer Lett..

[14] Hood JL, San RS, Wickline SA (2011). Exosomes released by melanoma cells prepare sentinel lymph nodes for tumor metastasis. Cancer Res..

[15] Shu SL (2018). Metabolic reprogramming of stromal fibroblasts by melanoma exosome microRNA favours a pre-metastatic microenvironment. Sci Rep..

[16] Lin LY (2016). Tumour cell-derived exosomes endow mesenchymal stromal cells with tumour-promotion capabilities. Oncogene..

[17] Peinado H (2012). Melanoma exosomes educate bone marrow progenitor cells toward a pro-metastatic phenotype through MET. Nat Med..

[18] Zhou X (2018). Melanoma cell-secreted exosomal miR-155-5p induce proangiogenic switch of cancer-associated fibroblasts via SOCS1/JAK2/STAT3 signaling pathway. J Exp Clin Cancer Res..

[19] Hood JL (2016). Melanoma exosome induction of endothelial cell GM-CSF in pre-metastatic lymph nodes may result in different M1 and M2 macrophage mediated angiogenic processes. Med Hypotheses..

[20] Boussadia Z (2018). Acidic microenvironment plays a key role in human melanoma progression through a sustained exosome mediated transfer of clinically relevant metastatic molecules. J Exp Clin Cancer Res..

[21] Wozniak M, Peczek L, Czernek L, Düchler M (2017). Analysis of the miRNA Profiles of Melanoma Exosomes Derived Under Normoxic and Hypoxic Culture Conditions. Anticancer Res..

[22] Cesi G (2018). A new ALK isoform transported by extracellular vesicles confers drug resistance to melanoma cells. Mol Cancer..

[23] Théry C (2018). Minimal information for studies of extracellular vesicles 2018 (MISEV2018): a position statement of the International Society for Extracellular Vesicles and update of the MISEV2014 guidelines. J Extracell Vesicles..

[24] König L (2017). Elevated levels of extracellular vesicles are associated with therapy failure and disease progression in breast cancer patients undergoing neoadjuvant chemotherapy. Oncoimmunology..

[25] Osti D (2019). Clinical Significance of Extracellular Vesicles in Plasma from Glioblastoma Patients. Clin Cancer Res..

[26] Shaked Y (2016). Balancing efficacy of and host immune responses to cancer therapy: the yin and yang effects. Nat Rev Clin Oncol..

[27] Keklikoglou I (2019). Chemotherapy elicits pro-metastatic extracellular vesicles in breast cancer models. Nat Cell Biol..

[28] Lee S (2018). Immunogenic Effect of Hyperthermia on Enhancing Radiotherapeutic Efficacy. Int J Mol Sci..

[29] Mahmood J (2018). Immunotherapy, Radiotherapy, and Hyperthermia: A Combined Therapeutic Approach in Pancreatic Cancer Treatment. Cancers (Basel)..

[30] Tallósy SP (2016). Adhesion and inactivation of Gram-negative and Gram-positive bacteria on photoreactive TiO_2_/polymer and Ag–TiO_2_/polymer nanohybrid films. Appl Surf Sci..

[31] Tallósy SP (2014). Investigation of the antibacterial effects of silver-modified TiO_2_ and ZnO plasmonic photocatalysts embedded in polymer thin films. Environ Sci Pollut Res Int..

[32] Szweda P (2015). Essential Oils, Silver Nanoparticles and Propolis as Alternative Agents Against Fluconazole Resistant *Candida albicans, Candida glabrata* and *Candida krusei* Clinical Isolates. Indian J Microbiol..

[33] Ahamed M, Khan MAM, Akhtar MJ, Alhadlaq HA, Alshamsan A (2017). Ag-doping regulates the cytotoxicity of TiO_2_ nanoparticles via oxidative stress in human cancer cells. Sci Rep..

[34] Tani F (2009). Surface expression of a C-terminal alpha-helix region in heat shock protein 72 on murine LL/2 lung carcinoma can be recognized by innate immune sentinels. Mol Immunol..

[35] Veres Á (2012). Silver and gold modified plasmonic TiO_2_ hybrid films for photocatalytic decomposition of ethanol under visible light. Catal Today..

[36] Hawley Robert J., Kozlovac Joseph P. (2005). Decontamination. Biological Weapons Defense.

[37] Kucharzewska Paulina, Belting Mattias (2013). Emerging roles of extracellular vesicles in the adaptive response of tumour cells to microenvironmental stress. Journal of Extracellular Vesicles.

[38] King HW, Michael MZ, Gleadle JM (2012). Hypoxic enhancement of exosome release by breast cancer cells. BMC Cancer..

[39] Parolini I (2009). Microenvironmental pH is a key factor for exosome traffic in tumor cells. J Biol Chem..

[40] Hedlund M, Nagaeva O, Kargl D, Baranov. V, Mincheva-Nilsson L (2011). Thermal- and oxidative stress causes enhanced release of NKG2D ligand-bearing immunosuppressive exosomes in leukemia/lymphoma T and B cells.. PLoS One.

[41] Jelonek K, Widlak P, Pietrowska M (2016). The Influence of Ionizing Radiation on Exosome Composition, Secretion and Intercellular Communication. Protein Pept Lett..

[42] Lv LH (2012). Anticancer drugs cause release of exosomes with heat shock proteins from human hepatocellular carcinoma cells that elicit effective natural killer cell antitumor responses *in vitro*. J Biol Chem..

[43] Yang Y, Chen Y, Zhang F, Zhao Q, Zhong H (2015). Increased anti-tumour activity by exosomes derived from doxorubicin-treated tumour cells via heat stress. Int J Hyperthermia..

[44] Harmati M (2017). Stressors alter intercellular communication and exosome profile of nasopharyngeal carcinoma cells. J Oral Pathol Med..

[45] Zapata-Benavides P (2012). WT1 silencing by RNAi synergizes with chemotherapeutic agents and induces chemosensitization to doxorubicin and cisplatin in B16F10 murine melanoma cells. Oncology letters..

[46] Veres Á (2014). Photocatalytic performance of silver-modified TiO_2_ embedded in poly(ethyl-acrylate-co-methyl metacrylate) matrix. Colloid Polym Sci..

[47] Mutschelknaus L (2017). Radiation alters the cargo of exosomes released from squamous head and neck cancer cells to promote migration of recipient cells. Sci Rep..

[48] Wozniak M, Peczek L, Czernek L, Düchler M (2017). Analysis of the miRNA Profiles of Melanoma Exosomes Derived Under Normoxic and Hypoxic Culture Conditions. Anticancer Res..

[49] Lazar I (2015). Proteome characterization of melanoma exosomes reveals a specific signature for metastatic cell lines. Pigment Cell Melanoma Res..

[50] Warburg O (1956). On the origin of cancer cells. Science.

[51] Vazquez A, Liu J, Zhou Y, Oltvai ZN (2010). Catabolic efficiency of aerobic glycolysis: the Warburg effect revisited. BMC Syst Biol..

[52] Hessvik NP, Llorente A (2018). Current knowledge on exosome biogenesis and release. Cell Mol Life Sci..

[53] Alaseem A (2019). Matrix Metalloproteinases: A challenging paradigm of cancer management. Semin Cancer Biol..

[54] Jabłońska-Trypuć A, Matejczyk M, Rosochacki S (2016). Matrix metalloproteinases (MMPs), the main extracellular matrix (ECM) enzymes in collagen degradation, as a target for anticancer drugs. J Enzyme Inhib Med Chem..

[55] Zheng Q, Ye J, Cao J (2014). Translational regulator eIF2α in tumor. Tumour Biol..

[56] Tai YL, Chen KC, Hsieh JT, Shen TL (2018). Exosomes in cancer development and clinical applications. Cancer Sci..

[57] Saleem SN, Abdel-Mageed AB (2014). Tumor-derived exosomes in oncogenic reprogramming and cancer progression. Cell Mol Life Sci..

[58] Becker A (2016). Extracellular Vesicles in Cancer: Cell-to-Cell Mediators of Metastasis. Cancer Cell.

[59] Penning TM, Drury JE (2007). Human aldo-keto reductases: Function, gene regulation, and single nucleotide polymorphisms. Arch Biochem Biophys..

[60] Tammali R, Reddy AB, Srivastava SK, Ramana KV (2011). Inhibition of aldose reductase prevents angiogenesis *in vitro* and *in vivo*. Angiogenesis..

[61] Sharma M (2013). Developmental Competence of Buffalo (*Bubalus bubalis*) Pluripotent Embryonic Stem Cells Over Different Homologous Feeder Layers and the Comparative Evaluation with Various Extracellular Matrices. Int J Stem Cells..

[62] Toth T (2018). Environmental properties of cells improve machine learning-based phenotype recognition accuracy. Sci Rep..

[63] Baglio SR (2017). Blocking Tumor-Educated MSC Paracrine Activity Halts Osteosarcoma Progression. Clin Cancer Res..

[64] Cho JA, Park H, Lim EH, Lee KW (2012). Exosomes from breast cancer cells can convert adipose tissue-derived mesenchymal stem cells into myofibroblast-like cells. Int J Oncol..

[65] Yamashita A, Hakura A, Inoue H (1999). Suppression of anchorage-independent growth of human cancer cell lines by the drs gene. Oncogene.

[66] Tarasov V (2007). Differential regulation of microRNAs by p53 revealed by massively parallel sequencing: miR-34a is a p53 target that induces apoptosis and G1-arrest. Cell Cycle..

[67] Ji Q (2009). MicroRNA miR-34 inhibits human pancreatic cancer tumor-initiating cells. PLoS One..

[68] Zhao L, Liu W, Xiao J, Cao B (2015). The role of exosomes and “exosomal shuttle microRNA” in tumorigenesis and drug resistance. Cancer Lett..

[69] Bar-On O, Shapira M, Hershko DD (2007). Differential effects of doxorubicin treatment on cell cycle arrest and Skp2 expression in breast cancer cells. Anticancer Drugs..

[70] Lüpertz R, Wätjen W, Kahl R, Chovolou Y (2010). Dose- and time-dependent effects of doxorubicin on cytotoxicity, cell cycle and apoptotic cell death in human colon cancer cells. Toxicology..

[71] Hibino S (2004). Identification of an active site on the laminin alpha5 chain globular domain that binds to CD44 and inhibits malignancy. Cancer Res..

[72] Lee DJ (2013). Peroxiredoxin-2 represses melanoma metastasis by increasing E-Cadherin/β-Catenin complexes in adherens junctions. Cancer Res..

[73] Stampolidis P, Ullrich A, Iacobelli S (2015). LGALS3BP, lectin galactoside-binding soluble 3 binding protein, promotes oncogenic cellular events impeded by antibody intervention. Oncogene..

[74] Friedl P, Wolf K (2003). Tumour-cell invasion and migration: diversity and escape mechanisms. Nat Rev Cancer..

[75] Huang Z, Yang M, Li Y, Yang F, Feng Y (2018). Exosomes Derived from Hypoxic Colorectal Cancer Cells Transfer Wnt4 to Normoxic Cells to Elicit a Prometastatic Phenotype. Int J Biol Sci..

[76] Lamalice L, Le Boeuf F, Huot J (2007). Endothelial cell migration during angiogenesis. Circ Res..

[77] Pan S (2008). Extracellular Alix regulates integrin-mediated cell adhesions and extracellular matrix assembly. EMBO J..

[78] Piccinini F, Santis I, Bevilacqua A (2018). Advances in cancer modeling: fluidic systems for increasing representativeness of large 3D multicellular spheroids. Biotechniques..

[79] Piccinini F (2015). AnaSP: a software suite for automatic image analysis of multicellular spheroids. Comput Methods Programs Biomed..

[80] Crawford S, Diamond D, Brustolon L, Penarreta R (2010). Effect of increased extracellular Ca^++^ on microvesicle production and tumor spheroid formation. Cancer Microenviron..

[81] Huang BW, Gao JQ (2018). Application of 3D cultured multicellular spheroid tumor models in tumor-targeted drug delivery system research. J Control Release..

[82] Samuel P (2018). Cisplatin induces the release of extracellular vesicles from ovarian cancer cells that can induce invasiveness and drug resistance in bystander cells. Philos Trans R Soc Lond B Biol Sci..

[83] Santos JC (2018). Exosome-mediated breast cancer chemoresistance via miR-155 transfer. Sci Rep..

