# Supplementary material for: Small extracellular vesicles convey the stress-induced adaptive responses of melanoma cells
Source: Sci Rep. 2019 Oct 25;9:15329. doi: 10.1038/s41598-019-51778-6 (PMC6814750; doi:10.1038/s41598-019-51778-6)
Supplement: Supplementary file 1 — Supplementary information [file 41598_2019_51778_MOESM1_ESM.pdf]

SUPPLEMENTARY INFORMATION

Small extracellular vesicles convey the stress-induced adaptive responses of melanoma cells

Maria Harmati[1,2], Edina Gyukity-Sebestyen[1,2], Gabriella Dobra[1,2], Laszlo Janovak[3], Imre Dekany[3], Okay Saydam[4], Eva Hunyadi-Gulyas[5], Istvan Nagy[6], Attila Farkas[7], Tibor Pankotai[8,9], Zsuzsanna Ujfaludi[8,9], Peter Horvath[1,10], Filippo Piccinini[11], Maria Kovacs[1], Tamas Biro[12,13], Krisztina Buzas[1,9]

[1]Laboratory of Microscopic Image Analysis and Machine Learning, Institute of Biochemistry, Biological Research Centre of the Hungarian Academy of Sciences, Szeged, Hungary
[2]Doctoral School of Interdisciplinary Medicine, Faculty of Medicine, University of Szeged, Szeged, Hungary
[3]Interdisciplinary Excellence Centre, Department of Physical Chemistry and Materials Science, University of Szeged, Szeged, Hungary
[4]Department of Pediatrics, University of Minnesota, Minneapolis, USA
[5]Laboratory of Proteomics Research, Institute of Biochemistry, Biological Research Centre of the Hungarian Academy of Sciences, Szeged, Hungary
[6]Sequencing Platform, Institute of Biochemistry, Biological Research Centre of the Hungarian Academy of Sciences, Szeged, Hungary
[7]Laboratory of Microbial Genomics, Institute of Plant Biology, Biological Research Centre of the Hungarian Academy of Sciences, Szeged, Hungary
[8]Department of Biochemistry and Molecular Biology, Faculty of Science and Informatics, University of Szeged, Szeged, Hungary
[9]Department of Oral Biology and Experimental Dental Research, Faculty of Dentistry, University of Szeged, Szeged, Hungary
[10]Institute for Molecular Medicine Finland, University of Helsinki, Helsinki, Finland
[11]Laboratorio di Bioscienze, Istituto Scientifico Romagnolo per lo Studio e la Cura dei Tumori (IRST) IRCCS, Meldola, Italy
[12]Department of Immunology, Faculty of Medicine, University of Debrecen, Debrecen, Hungary
[13]Hungarian Centre of Excellence for Molecular Medicine, Szeged, Hungary

SUPPLEMENTARY METHODS

Proteomic analysis by LC-MS/MS

Separation and in-gel digestion of sEV proteins

Vesicular pellets were resuspended in NP40 lysis buffer (Invitrogen) and incubated on ice for 30 min. Protein content of sEV isolates were measured as described in the Methods section and 25 µg protein of each sample was diluted in a mixture of 4× sample buffer (NuPAGE LDS Sample Buffer) and 10× reducing agent (NuPAGE Sample Reducing Agent), boiled at 96°C for 10 min, immediately cooled on ice and separated in 4-12% Bis-Tris Protein Gel (NuPAGE Novex) using an XCell SureLock Mini-Cell (Invitrogen) at 200 V and 0.03 A for 40 min with an electrophoresis buffer (NuPAGE MOPS SDS Running Buffer). Then, the gel was stained overnight with Coomassie blue (0.1% Coomassie Brilliant Blue R-250 in 45% ethanol, 45% MQ water and 10% acetic acid), destained with a mixture of 10% acetic acid, 30% ethanol and 60% MQ water and washed in MQ water. Each lane was cut to 12 equal bands and subjected to in-gel digestion. Gel bands were diced to smaller pieces, and the SDS and the dye were washed out with 3× 50 µl 25mM ammonium-bicarbonate (ABC)/50% Acetonitrile (AcN). After reduction with DTT (1,4-dithiothreitol, Sigma; 20 µl, 10 mM DTT in 25 mM ABC) at 56°C for 30 min, and alkylation with IAM (iodoacetamide, Sigma; 20 µl, 55 mM IAM in 25 mM ABC) at room temperature (RT) in dark for 30 min, the gel samples were dried in a vacuum centrifuge and after that rehydrated in 20 µl of trypsin (Sequencing Grade Modified Trypsin, Promega; 5 ng/µl in 25 mM ABC) and incubated at 37°C. The digestion was stopped after 4 h by lowering the pH of the buffer below 3, by adding 2 µl of 10% formic acid (FA). Tryptic peptides were extracted from the gel with 3× 50 µl of 2% FA in 50% AcN and dried. Prior mass spectrometric analysis, all samples were redissolved in 50 µl of 0.1% FA.

Mass spectrometry analysis and protein identification

Samples were analysed on an LTQ-Orbitrap Elite (Thermo Scientific) mass spectrometer on-line coupled with a nanoHPLC (nanoAcquity, Waters) system. Five µl of the in-gel digests were loaded (for 3 min at 8 µl/min flow, using 0.1% FA in 3% AcN-97% water) onto a reversed phase trap column (Waters, Symmetry C18, 0.18x20 mm) and separated on a C18 reversed phase (Waters, BEH300C18 1.7 µm) nanocolumn (0.075x200 mm). The flow rate was 330 nl/min and a linear gradient was used from 3% to 40% solvent B in 37 min (solvent A was 0.1% FA in water and solvent B was 0.1% FA in AcN).

The high voltage (1.2 kV) was applied through the liquid junction between the chromatographic column and the non-coated silica nanospray emitter (NewObjective, 10 µm tip ID). The mass spectrometer operated in data-dependent mode: the survey mass spectra were detected in the Orbitrap with high resolution (R=60k @ m/z: 400, mass range m/z: 380-1400) and the most abundant multiply charged 20 peaks were selected for ion-trap fragmentation (NCE: 35%; activation q: 0.25; activation time: 10 ms; minimum signal intensity: 5000 counts). The MS/MS spectra were detected in the ion trap. Dynamic exclusion was used, the precursors were excluded for 15 s after the first fragmentation event.

Data analysis: searchable peaklists (mgf format) were extracted using Proteome Discoverer 1.4 (Thermo Scientific) and subjected to database search on our in-house ProteinProspector 5.14.1 search engine using the following parameters: parent ion tolerant: 5 ppm; fragment ion tolerant: 0.6 Da; Cys carbamidomethylation was set as constant and Met oxidation, cyclisation of peptide N-terminal Glu to pyroglutamic acid, protein N terminal acetylation were set as variable modifications. Only fully tryptic peptides were considered with maximum of 2 missed cleavage sites. The *Mus musculus* and *Bos taurus* protein sequences of the Uniprot (UniProtKB.06.11.2014) database completed with human keratins and pig trypsin, altogether 106,330 protein sequences were searched.

For the false discovery rate (FDR) estimation, the searches were performed on the database concatenated with the randomized sequences. Protein identification was accepted if the ProteinProspector expectation value was less than 0.01 and the protein was identified with at least 3 unique peptides (expectation value less than 0.05 and score higher than 15). Peptides with identical bovine and mouse sequence were excluded also. FDR values were less than 1% in all cases.

Western blot

Protein samples were prepared and separated beside a ProSieve color protein marker (Lonza), as described above. Then proteins were transferred to an Immobilon-P PVDF membrane (Millipore) using an XCell SureLock Mini-Cell (Invitrogen) at 30 V and 10-170 mA for 60 min with a transfer buffer (NuPAGE). Blocking was performed by 5% non-fat milk in TBST buffer (25 mM Tris-HCl, 150 mM NaCl, 0.05% Tween-20, pH 7.2) for 60 min at RT. For detection of the vesicular markers, we used rabbit anti-mouse CD9 (1:500, LifeSpan Biosciences), rabbit anti-mouse CD63 (1:500, Santa Cruz), mouse HSP70/HSP72 monoclonal antibody (1:8000, Enzo Life Sciences, Inc.), rabbit anti-mouse Alix (1:500, Sigma-Aldrich), rabbit anti-mouse TSG101 (1:2500, Sigma-Aldrich), rabbit anti-mouse Calnexin (1:1000, Abcam) and rabbit MLANA polyclonal antibody (1:2000, ABclonal). Primary antibody labelling was performed in 1% non-fat milk in TBST buffer overnight at 4°C. Unbound antibodies were removed by washing 3 times for 10 min in TBST buffer. As secondary antibodies, HRP-conjugated anti-rabbit IgG and anti-mouse IgG (R&D Systems) were used in 1% non-fat milk-containing TBST buffer with 60 min incubation at RT. After washing, protein bands were visualised by the WesternBright ECL detection kit (Advansta) and an Odyssey Fc imaging system (LI-COR) coupled with the Image Studio Lite 5.2 software.

AFM

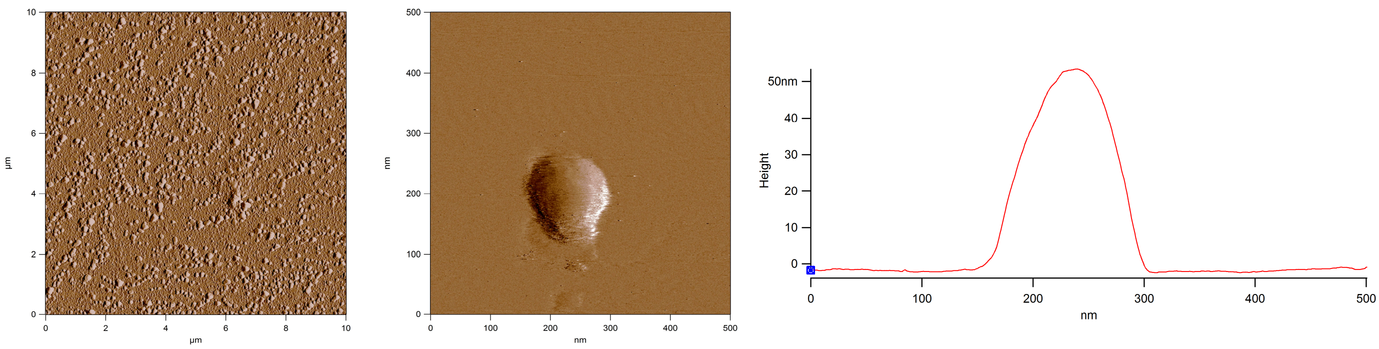

DLS by Zetasizer Nano S

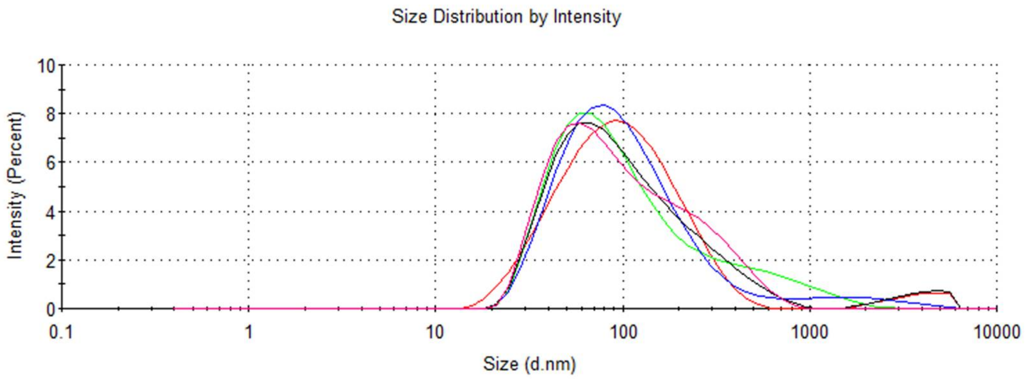

	Z-Average (d.nm)
Mean	78.27
SD	0.7796

Western blot

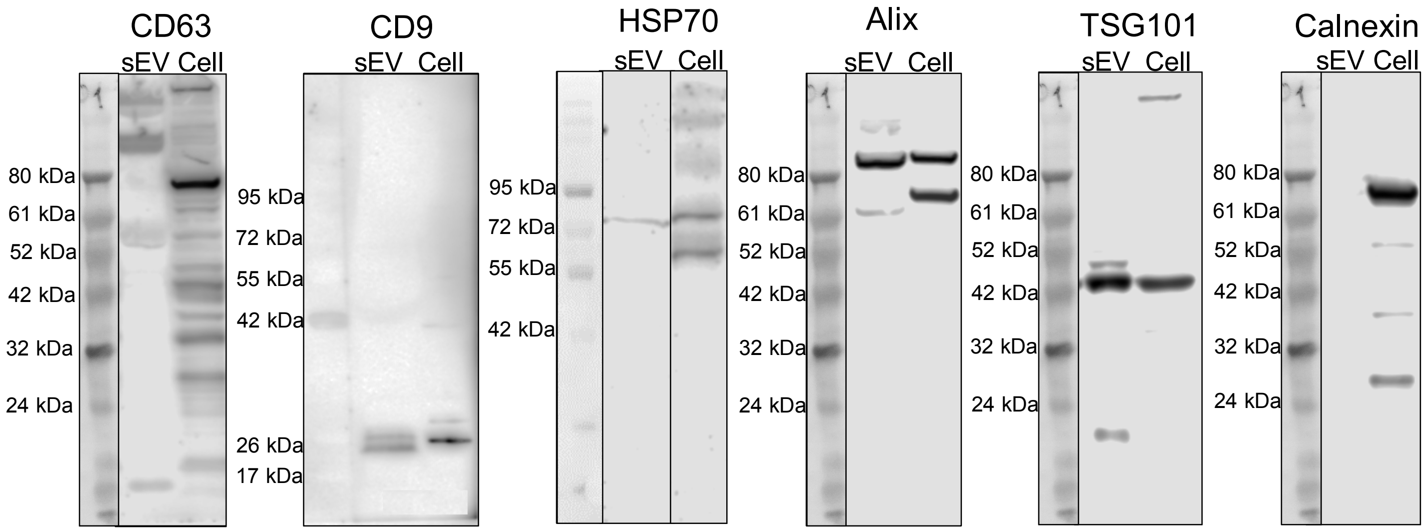

Supplementary Figure S1. Isolated B16F1 cell-derived vesicles showed exosomal characteristics. Spherical shape and small size of sEVs were verified by AFM (top row). Size distribution of the isolated sEV population was analysed by DLS using a Zetasizer Nano S instrument (middle row). EV markers such as CD63 and CD9 (transmembrane proteins), HSP70, Alix and TSG101 (cytosolic proteins), Calnexin (negative sEV marker) were investigated in the vesicle isolates and the donor cell lysates by Western blot (bottom row). sEV and Cell samples were investigated on the same gels/blots for each marker, however their lines were cropped, shown by black boxes.

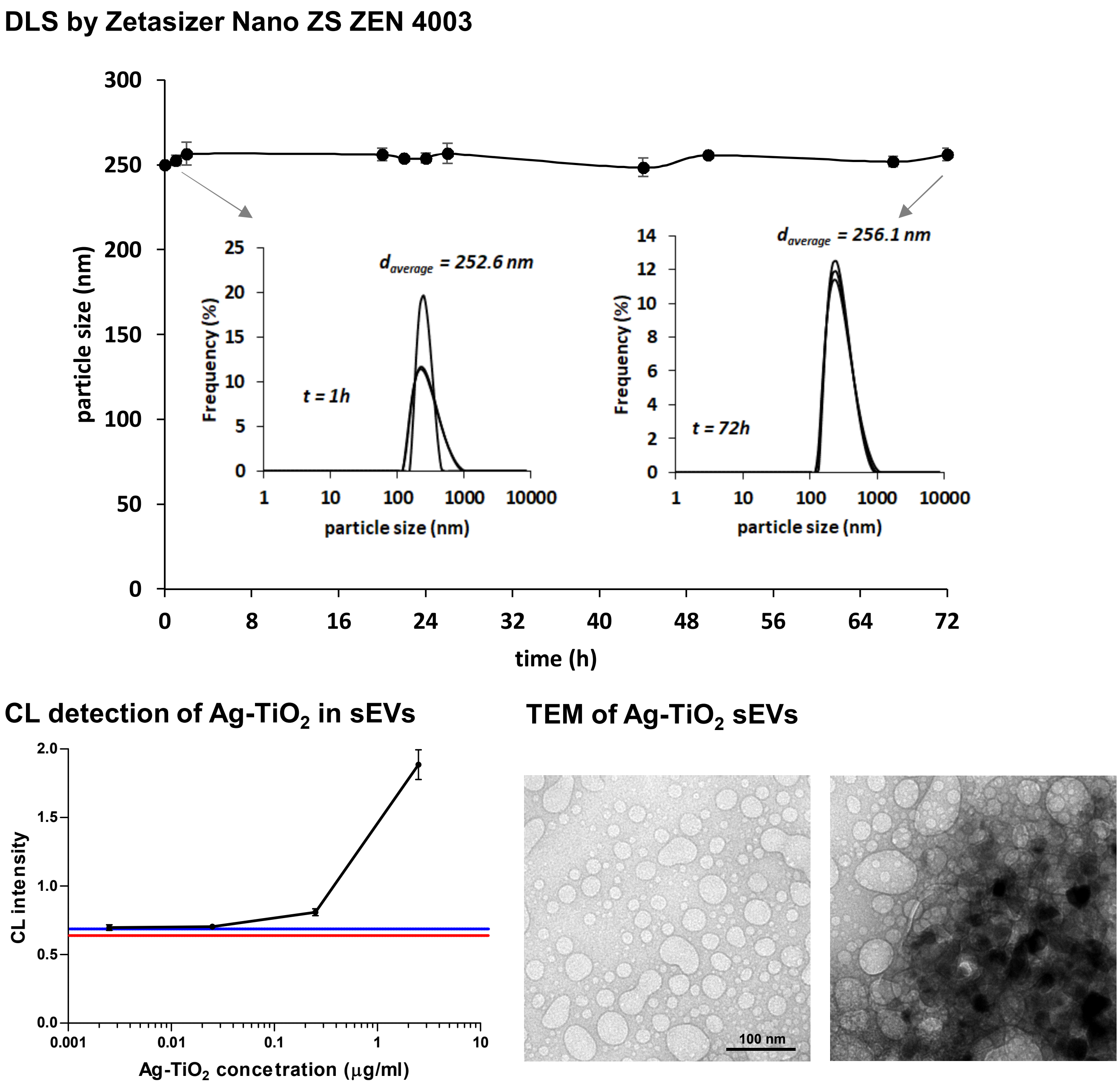

Supplementary Figure S2. Ag-TiO_2 transfer by sEVs cannot be proven.

Upper diagram shows the size distribution of the Ag-TiO_2 photocatalyst particles, which was around 255 nm during the whole time interval of stress exposures. Measurements were performed by DLS, figure shows the measured average hydrodynamic particle diameter values of Ag-TiO_2 particles in B16F1 culture medium, as a function of time. The inserted figures show the size distribution curves measured at 1 h and 72 h, respectively.

Lower diagram shows the result of the Ag-TiO_2 detection using chemiluminescence method. Black line is the Ag-TiO_2 calibration curve (each point represent mean±SD values). Blue and red lines show the mean CL intensities of the Ag Ctrl sEV (background) and Ag-TiO_2 sEV lysates, respectively.

Left TEM image shows the Ag-TiO_2 sEVs, which do not contain inner structures, including electrondense Ag-TiO_2 nanoparticles. Right TEM image shows EVs mixed with Ag-TiO_2 nanoparticles as a reference. Cause of the high concentration of vesicles and nanoparticles, they are seen above each other.

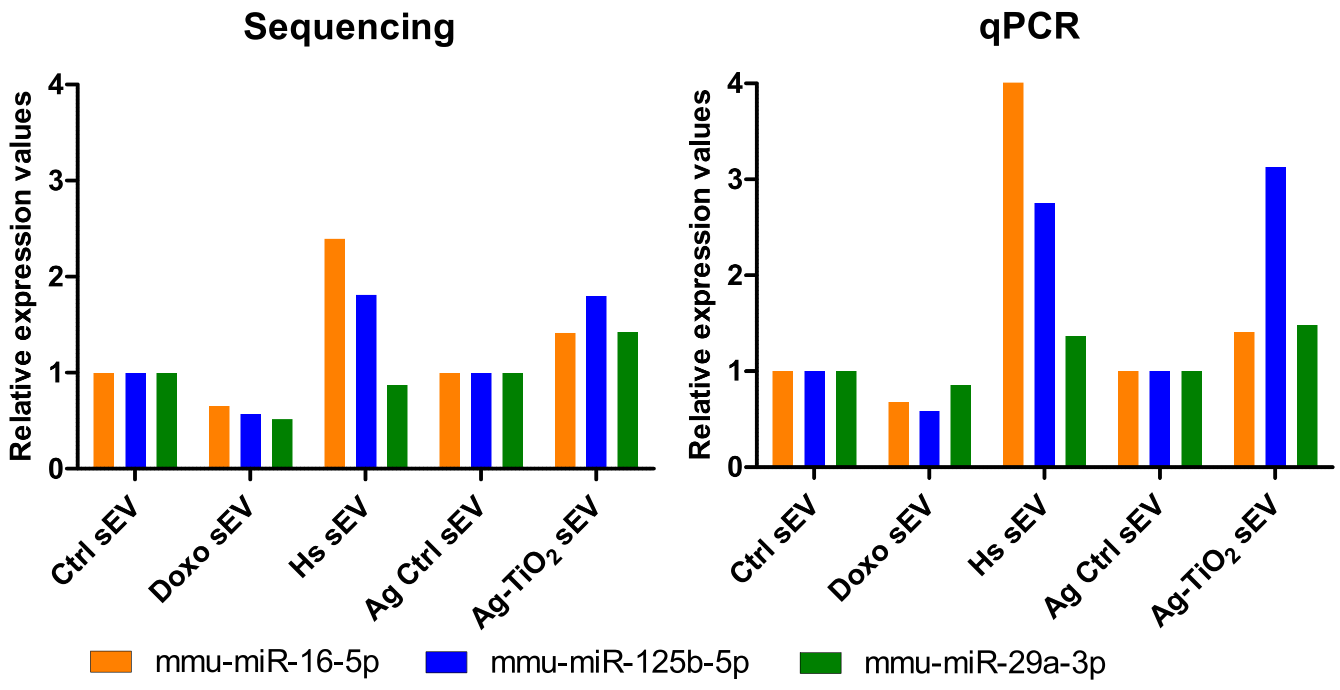

Supplementary Figure S3. Validation of the miRNA sequencing results by qPCR. Relative expression values of mmu-miR-16-5p, mmu-miR-125b-5p and mmu-miR-29a-3p showed similar patterns for both quantification methods.

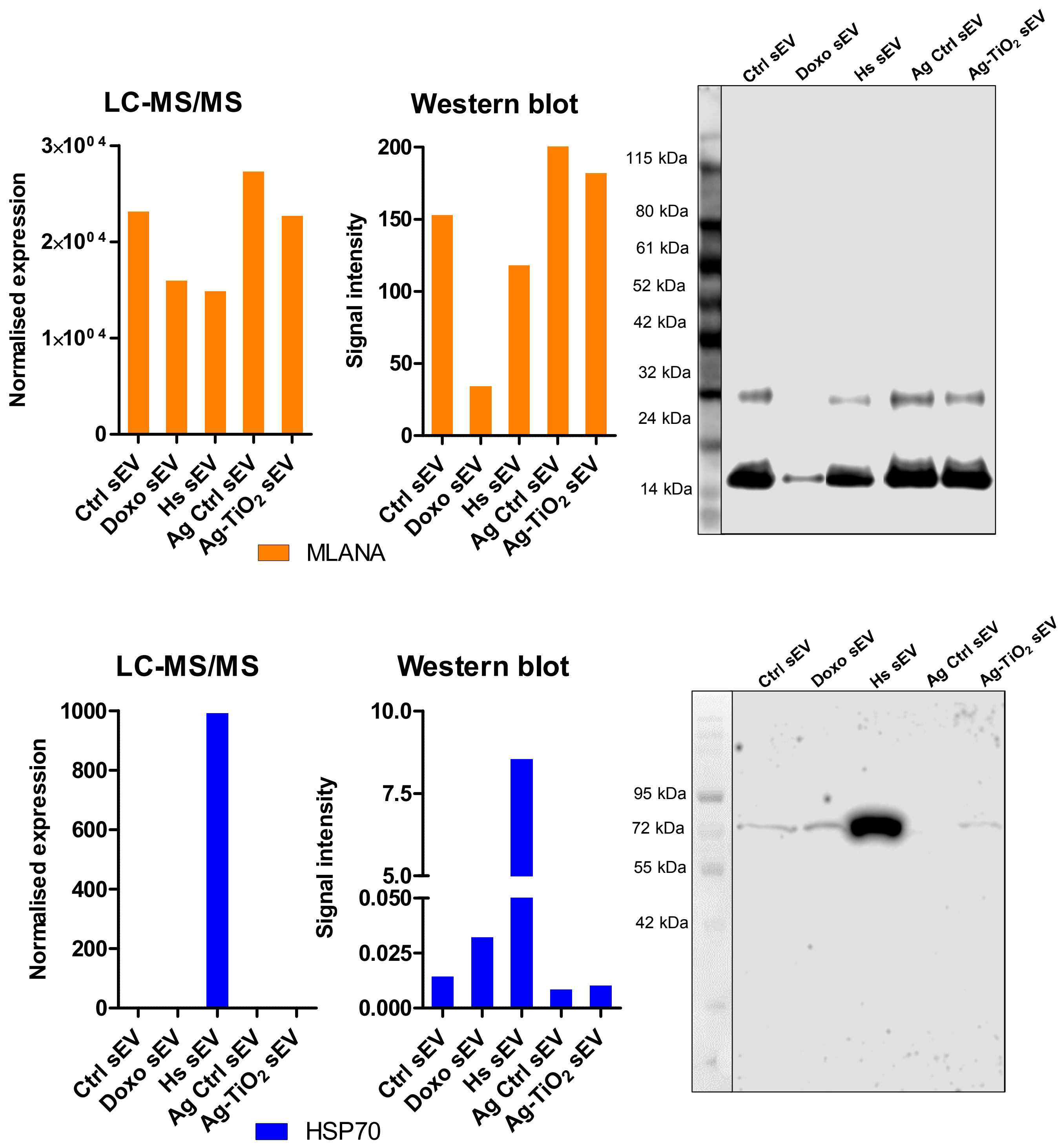

Supplementary Figure S4. Validation of the LC-MS/MS results by Western blot. Signal intensities of HSP70 and MLANA were quantified by densitometry, which reflected the LC-MS/MS resulted differences between the sEV groups. However, using Western blot, which is a more sensitive method, we were able to detect HSP70 in each sEV isolate. sEV and Cell samples were investigated on the same gels/blots for each marker, however their lines were cropped, shown by black boxes.

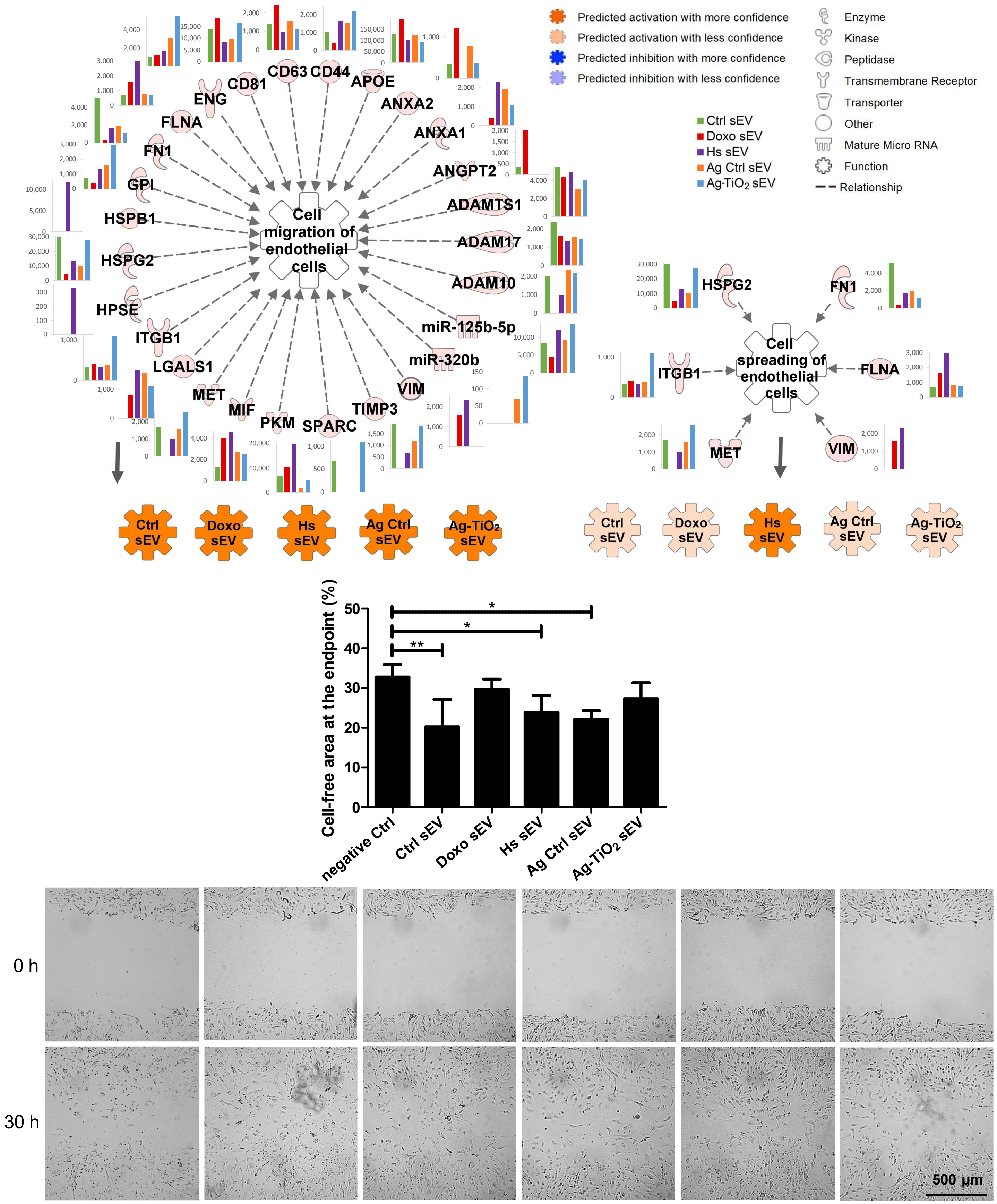

Supplementary Figure S5. Melanoma sEVs facilitate the endothelial cell migration. Networks show the IPA predictions for the regulatory effects of sEV molecules on the 'Cell migration of endothelial cells' and ‚Cell spreading of endothelial cells'. Networks show every upstream regulator proteins and miRNAs accompanied by a bar graph, which represents the normalised expression values of the molecule for each sEV group. Coloured symbols, named as the sEV groups, display the expected regulation changes of the analysed 'Biofunctions' upon exposure to the vesicles. Bar graph shows the result of the analysis of wound closures by the ImageJ wound healing tool. It shows mean+SD values (n=4). Images represent the wounds after 30 h of sEV exposures.

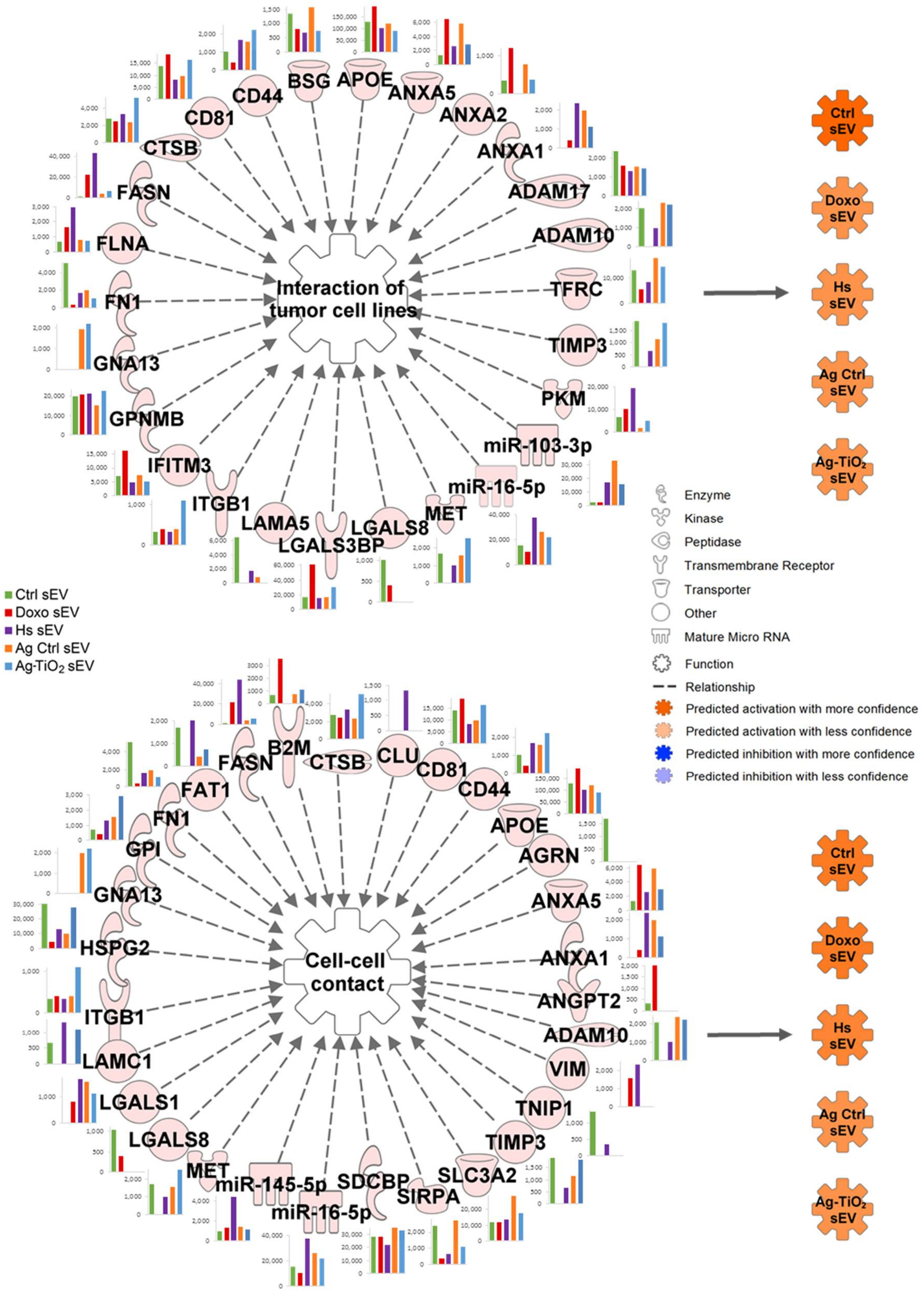

Supplementary Figure S6. IPA predictions for regulatory effects of sEV molecules on the 'Interaction of tumour cell lines' and the 'Cell-cell contact' showed activations. Networks show every upstream regulator proteins and miRNAs accompanied by a bar graph, which represents the normalised expression values of the molecule for each sEV group. Coloured symbols, named as the sEV groups, display the expected regulation changes of the analysed 'Biofunctions' upon exposure to the vesicles.

Group name	negative Ctrl	Ctrl sEV	Doxo sEV	Hs sEV	Ag Ctrl sEV	Ag-TiO_2 sEV
Eq. diameter (µm)	437 ±9	351 ±15	309 ±16	423 ±5	347 ±18	348 ±17
Major diameter (µm)	519 ±17	489 ±20	388 ±23	530 ±20	448 ±30	432 ±32
Minor diameter (µm)	323 ±12	170 ±40	242 ±17	306 ±19	212 ±20	257 ±30
Convexity	0.662 ±0.037	0.632 ±0.017	0.730 ±0.064	0.585 ±0.050	0.690 ±0.165	0.630 ±0.069
Solidity	0.774 ±0.021	0.734 ±0.011	0.838 ±0.009	0.800 ±0.020	0.825 ±0.080	0.833 ±0.046
Sphericity	0.554 ±0.042	0.501 ±0.013	0.629 ±0.042	0.491 ±0.034	0.601 ±0.180	0.547 ±0.066

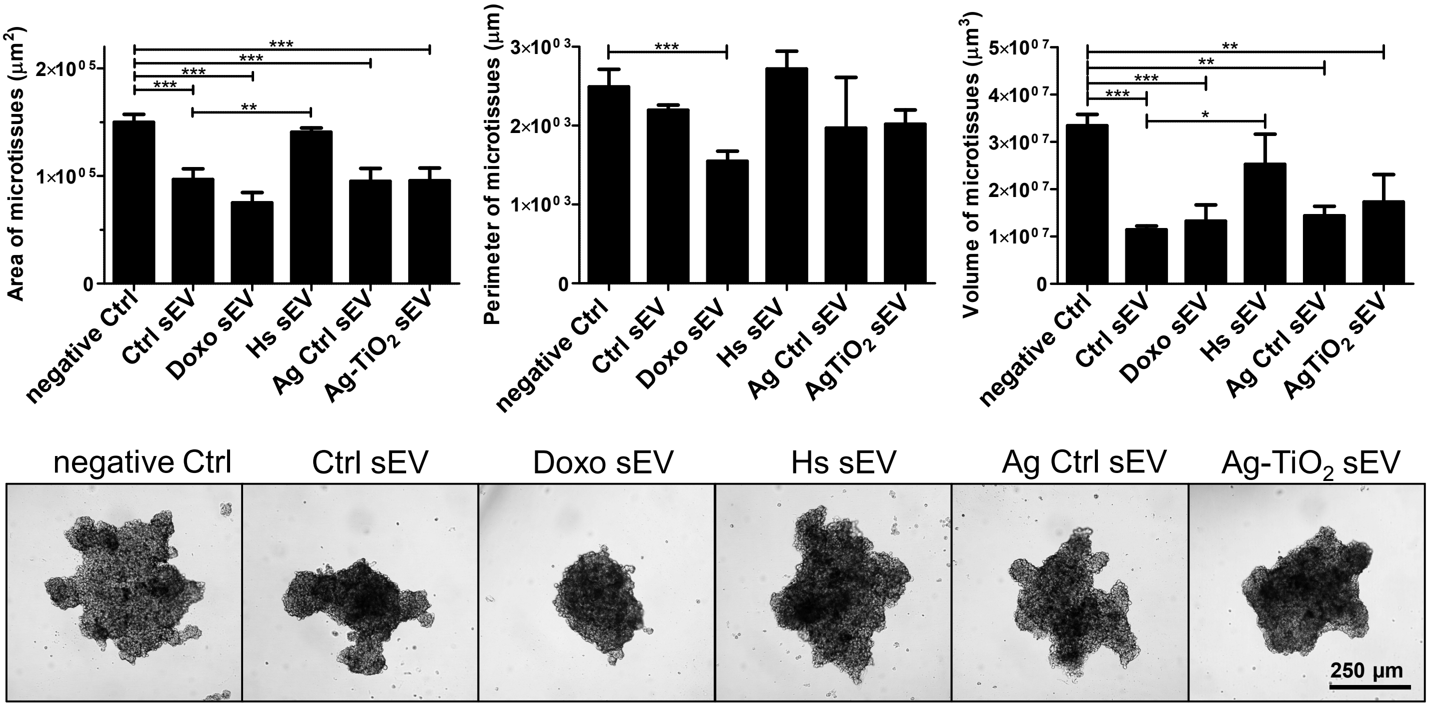

Supplementary Figure S7. Validation of the microtissue generation promoting effects of sEVs using MEF-B16F1 3D co-cultures.

Table shows the statistics about the calculated diameters and compactness using the AnaSP software. Eq. diameter means equivalent diameter, major and minor diameters are measured through centroid. Table contains mean±SD values.

Bar graphs show the area, perimeter and volume statistics of the generated microtissues (mean+SD, n=3). Statistical evaluation was performed by Welch's ANOVA test with Tukey's HSD post-hoc test; *p<0.05, **p<0.01 and ***p<0.001 indicate statistical significance.

Images represent the generated microtissues after 72 h of sEV exposures.
